# Population-Area Relationship for Medieval European Cities

**DOI:** 10.1371/journal.pone.0162678

**Published:** 2016-10-05

**Authors:** Rudolf Cesaretti, José Lobo, Luís M. A. Bettencourt, Scott G. Ortman, Michael E. Smith

**Affiliations:** 1 School of Human Evolution and Social Change, Arizona State University, Tempe, Arizona, 85281, United States of America; 2 School of Sustainability, Arizona State University, Tempe, Arizona, 85281, United States of America; 3 Santa Fe Institute, 1399 Hyde Park Rd, Santa Fe, New Mexico, 87501, United States of America; 4 Department of Anthropology, University of Colorado Boulder, Boulder, Colorado, 80309, United States of America; University of Lausanne, SWITZERLAND

## Abstract

Medieval European urbanization presents a line of continuity between earlier cities and modern European urban systems. Yet, many of the spatial, political and economic features of medieval European cities were particular to the Middle Ages, and subsequently changed over the Early Modern Period and Industrial Revolution. There is a long tradition of demographic studies estimating the population sizes of medieval European cities, and comparative analyses of these data have shed much light on the long-term evolution of urban systems. However, the next step—to systematically relate the population size of these cities to their spatial and socioeconomic characteristics—has seldom been taken. This raises a series of interesting questions, as both modern and ancient cities have been observed to obey area-population relationships predicted by settlement scaling theory. To address these questions, we analyze a new dataset for the settled area and population of 173 European cities from the early fourteenth century to determine the relationship between population and settled area. To interpret this data, we develop two related models that lead to differing predictions regarding the quantitative form of the population-area relationship, depending on the level of social mixing present in these cities. Our empirical estimates of model parameters show a strong densification of cities with city population size, consistent with patterns in contemporary cities. Although social life in medieval Europe was orchestrated by hierarchical institutions (e.g., guilds, church, municipal organizations), our results show no statistically significant influence of these institutions on agglomeration effects. The similarities between the empirical patterns of settlement relating area to population observed here support the hypothesis that cities throughout history share common principles of organization that self-consistently relate their socioeconomic networks to structured urban spaces.

## Introduction

Scholars have long debated the role of the medieval city in the long-term economic development of Europe. As concentrations of population in space, medieval urban areas are recognizable as “cities”, in a modern sense. They were also centers of commerce, manufacture, and innovation, possessed long-range trade networks, and had recognizable divisions of labor [1–9]. Indeed, many scholars have maintained that the organizational, social, political, and economic innovations that would usher in “modernity,” “capitalism,” and the Industrial Revolution originated in medieval European settlements [10–27]. At the same time, others have argued a qualitative watershed separates medieval from industrial cities [1,25,28–32], and for a series of crucial changes during the Early Modern period and Industrial Revolution [6,12,19,33–42]. To be sure, medieval cities were much smaller. Situated within agrarian societies, productivity was lower, technology simpler, and the market economy much less developed [6,11,36,43–47]. Church, crown, and guilds had a far greater influence on the regulation of social and economic life than in both more recent and contemporary urban systems [4–7,9,10,14,48–54]. Cast in this light, medieval cities appear structured, corporate societies where social groupings limited social and economic opportunities for individuals and households to a much greater extent than today. These two contrasting perspectives leave us with an empirical question: Were medieval European cities fundamentally different from industrial and modern cities? Or were they part of a general continuum of urban form and function?

Far from just a particularistic issue, answers to such questions are important for constructing a general, historically-informed theory of urbanization. Here, we utilize the emerging framework of *settlement scaling theory* to investigate one dimension of potential similarity between modern and medieval cities. Recent work has identified a number of statistical regularities in the properties of contemporary urban systems that reflect underlying socioeconomic processes of interaction and exchange. This research suggests that, at a fundamental level, cities consist of overlapping social and physical networks that are self-consistently bounded by settled physical space [55–57]. Here, we investigate whether the relationships between settlement population and settled land area predicted by scaling theory—and observed in contemporary cities—also characterized medieval European cities.

In this paper, we analyze the relationship between the extent of built-up area and resident populations of 173 settlements located in present-day Belgium, France, England, Switzerland, Germany, and Italy, *ca*. AD 1300. Previous scholarship has produced population estimates for a large number medieval European cities [58,59]. We build on this work by linking population estimates with estimates for the built-up area compiled from historical and archaeological sources (see “Materials and Methods” section, below).

To interpret these data, we introduce two formal models of settlement scaling as social networks embedded in space, which derive differing predictions for how the spatial organization of medieval cities and towns may vary with population size. The first model, which was originally developed to characterize modern cities [55], derives the built-up area of cities as a function of their population size given their essential characteristic as self-organizing, non-hierarchical socioeconomic networks of interactions imbedded in physical space [60,61]. The second model adds socioeconomic restrictions to the first model, based on potential impacts of medieval institutional structures on social networking. This second model predicts that, if such restrictive effects are strong, cities should show weak or vanishing agglomeration effects, and this should be apparent in the relationship between built area and population.

Next, we provide a brief overview of the socioeconomic characteristics of medieval European cities, address how these were articulated across space, and divide our sample of 173 settlements into historically valid regional urban systems. This sample of European cities is then evaluated against the population-area predictions of the two urban scaling models. We conclude by placing our results in the context of similar studies on ancient and modern cities, discussing potential consequences for our understanding of medieval urbanism in Europe, and identifying promising future work.

## Models of Social and Spatial Organization in Cities

Medieval cities provide an opportunity to discuss two seemingly disparate views of cities. On the one hand, one can imagine a city as a self-organized, non-centralized social and spatial arrangement. In this view, interactions among people are only impeded by transaction, transportation and opportunity costs, and the cost of matching any one individual’s resources, needs, interests and skills with those of another individual (known in the urban economics literature as “matching costs” [62]). Such networks are “well-mixed”, in the sense that all types of interactions between any two individuals are possible. This kind of model has a long pedigree in economics and geography and is described in some detail below. We will refer to this view of cities as the *social reactor model*.

But history provides reasons to consider urban agglomerations very differently. Medieval European settlements were controlled by a variety of hierarchically-organized social institutions such as political authorities, religious organizations, guilds, classes, and kinship groups operating across the urban system [1,3–6,19,63–65]. As noted in the introduction, this may lead to the hypothesis that such institutional structures strongly regulate the socioeconomic contacts between individuals—thus restricting the social interactions that generate economic relations, flows of ideas, and the joint creation of knowledge. Below we develop a mathematical model, based on hierarchical graphs, that captures the essence of this hypothesis. We will refer to this second view as the *structured social interaction model*.

Below, we introduce these two models and describe their main assumptions and relationship to previous urban theory. We also formulate their expectations in terms of scaling relations—specifically the relationship between population size and areal extension—which we use afterwards to investigate the nature of the medieval city in light of our data.

### The Social Reactor Model

The starting point of most socioeconomic theory of cities, including classical models of geography and urban planning [66], is the idea of a “spatial equilibrium”. Such equilibrium applies only to a short-term balance between costs and benefits of living in a city and thus admits flows of people and information between cities over longer time periods and their population and economic growth. Other classes of urban models, such as those of spatial morphogenesis based on local rules [67–69], assume spatial growth mechanisms that are more abstract and less directly connected to socioeconomic considerations and will not be discussed here.

The first quantitative models of spatial equilibrium and spatial agglomeration can be traced back to von Thünen, almost two centuries ago [70]. He did not actually consider a city, but a central market in which agricultural products with different values and transportation costs were to be sold. Von Thünen observed that heterogeneity of products in terms of their price and transportation costs naturally resulted in mathematically predictable heterogeneous land uses, with more valuable and perishable products taking up land closer to the central market and vice-versa.

This framework was subsequently transformed into a model for land rents in cities by Alonso in the 1960s [60]. The idea was that within a city, land rents vary across space and individuals and firms optimize their location by balancing rents against transportation costs and preferences, within a total budget. More sophisticated implementations of this model in urban economics [61,71] assume—like von Thünen—a monocentric circular symmetric city and preferences given by standard utility and production functions, as well as simple transportation costs proportional to distance.

Urban scaling theory, which we briefly outline below, and its development in the context of historical settlements [56] has the same initial ingredients as the Alonso model [55]. However, the scaling framework includes a series of structures and refinements that provide the resulting models with more general microscopic and physical foundations. As a result, scaling theory allow for the computation of scaling relations and the quantitative determination of exponents and prefactors (population independent terms) in regression equations relating different salient characteristics of cities to urban population size. It is these predictions that we test against data from medieval European cities in the following section.

Relative to models from urban economics, urban scaling theory elaborates on three major aspects of urban socioeconomic relations [55]: i) physical transportation costs, ii) the actual structure of the built space of cities and iii) socioeconomic networks as the basis for determining productivity (instead of assumed production functions). Although these aspects introduce greater heterogeneity—of social structure and socioeconomic outcomes, for example—scaling relations are derived as city-wide *averages* and thus can be computed as a function of just a few parameters [55,72]. While it is certainly true that cities always exhibit extreme spatial and social heterogeneity, together these diverse components perform functions only possible as an interacting social system [73]. In this way, global properties are especially valid metrics of a settlement because the ‘whole is greater than the sum of its parts.’ Predicting local variations across individuals and places within a city can also be achieved in this theoretical framework, but this requires more fine-grained data that are not even yet widely-available for modern cities [74].

A key difference between urban economics models and urban scaling theory is the replacement of a production function by a socioeconomic network of interactions [55,75]. While the use of a production function might be well justified when explicitly modeling the generation of economic outputs [61,76], the use of social networks to represent interactions among humans is a more general modeling framework, applicable to any human settlement throughout history [56].

The fundamental idea behind settlement scaling theory is that all human settlements—regardless of scale or social complexity—share fundamental quantitative similarities in terms of their overall form and function. This derives from the existence of system-wide advantages of social agglomeration, whether for defense, economic specialization, the generation of innovations, shared infrastructure, religion, innovation, or trade [77,78]. Other commonalities for urban systems embedded in different historical and geographic settings are restrictions in the use of land (which is a rival and excludable economic good) and the presence of transportation costs. In the following paragraphs we derive the basic expectations of this theory (More detailed derivations are given in Ref. [55]).

First, we derive the expected relationship between the population of a settlement, *N*, and its overall land area, *A*. This can be done without assuming that the city is radially symmetric, as in older models [60]. To do this we balance the average benefit that accrues to an individual from social interactions, *y* = *GN/A*, against the associated transportation costs, *c* = *εA*^*H/2*^. The first expression is derived by embedding a social network in space [55], where an individual meets others in a settlement at an average rate proportional to its density *N/A*, which is the expected rate of encounters [55]. The parameter *G* is the net benefit per interaction [79] Times the area swept by an individual over the given time. The nature of the social and economic interactions that an individual can experience—and therefore the strength and composition of *G*—is certainly context-dependent, and very different in a medieval vs. a modern city. However, such differences can be captured in a simple way on aggregate via changes in *G*. The per capita cost of realizing interactions, *c*, is set by the cost of movement per unit length *ε*, which is a function of technology (e.g. walking vs. horse-riding) and also of how individuals explore the space of a city, *A*, parameterized by a fractal dimension, *H*, for their trajectories. For *H* = 1, individuals can explore the city through a line-like trajectory, whereas as *H* → 2, individuals explore the city exhaustively as an area, which typically becomes difficult in larger places. In the limit *H* → 0, individuals stay limited to a single place (their “trajectory” is point-like) and the city falls apart as a socioeconomic network of interaction. To see this we equate benefits to costs and solve for area as a function of population to obtain

$$A\left(N\right)=a\;N^{\alpha },\text{ with }\alpha =\frac{2}{H+2},\;a={\left(\frac{G}{\varepsilon }\right)}^{\alpha }\cdot$$ (1)

This simple result already captures some of the most important characteristics of human settlements. First, if social benefits are small relative to transportation costs, which is likely true in medieval cities, then the prefactor, *a*, will be small and all settlements will be quite dense (we refer to the parameter *a* as the baseline area per person). We also see that if *H* = 0, as might occur in a segregated settlement, the total settled area becomes proportional to population and no agglomeration effects are present. For *H* = 1, one obtains the special value for the exponent, *α* = 2/3, a situation we described elsewhere [56] as the *amorphous settlement model*, because it neglects any structure in urban built space. In such settlements, population density, *n*, increases strongly with population size as $n\;\left(N\right)=\frac{N}{A\left(N\right)}=a^{-1}N^{1/3}$.

However, in deriving these properties we have not yet considered the fact that urban land use and built space become increasingly structured as settlements grow. Specifically, settlements become organized in terms of access networks (streets, canals, paths) and places, which include locations of work and residence as well as public spaces [80]. This leads to a characteristic topology of cities, set by the spatial relationship among places and access networks that characterizes all cities, regardless of their specific geometry [68]. This means that the relevant space for social interactions in cities is set by its *access network*. We designate the total area of this network *A*_*n*_, and compute it via a decentralized infrastructure network model, which predicts that the area of network per individual is set by the length scale derived from the density *n* (see [55]), as

$$A_{n}=l\;n^{-1/2}N=a_{0}N^{1-\delta },\text{ with }\delta =\frac{H}{2\left(H+2\right)},\;a_{0}=l\;a^{1/2},$$ (2)

where *l* is a length scale measuring the width of the network at each end-place (e.g. doors and entrance-ways). This calculation can also be derived from a more detailed model of infrastructure from which it can be shown that the costs of transportation over these networks, *W*, scales superlinearly with population size, *W*∼*Nc*∼*N*^1+*δ*^, just like socioeconomic interactions in built space, $Y=yN\sim G\frac{N^{2}}{A_{n}}\sim N^{1+\delta }$ [55]. This results in a new spatial equilibrium model for a *networked settlement*. The basic unit of settlement productivity is then the total number of socioeconomic interactions it sustains, *K*(*N*) = *k*_0_*N*^1+*δ*^∼*Y*(*N*) [56]. Such a model predicts many of the characteristics of modern cities [81], in broad agreement with empirical observations. It also applies to at least some settlement systems in antiquity, such as those of the Pre-Columbian Basin of Mexico and Central Andes [56,57,82].

Relative to models from urban economics, scaling theory has the advantage of not requiring the specification of production functions or utility functions accounting for the behavior of firms or consumers. Instead, increasing returns to scale in the productivity of cities—the fact that *Y* increases per capita with city size—derives from network effects and their scope for the specialization of knowledge and labor. In this way, scaling effects are a spatially limited version of Metcalfe’s law, where the value of a network is proportional to the number of possible connections [83]. A production function for settlements, showing increasing returns to scale, can then be derived as an emergent property of these socioeconomic networks embedded in the structured spaces of each settlement [76].

For the analysis carried out in the remainder of this paper, two properties of the *social reactor model* are especially important. First, this model predicts the settled area of a city or town in a given urban system should increase with its population, on average, with an exponent $\frac{2}{3}\leq \alpha \leq \frac{5}{6}$ where the lower range would correspond to amorphous settlements, typically small sites, and the upper value to the fine measurement of all built up surfaces in structured settlements.

The second property derives from the extent to which social interactions are structured by group affiliations. We turn to this issue next.

### The Structured Social Interaction Model

We now show how social groups and institutions can mediate social interactions and introduce further constraints to urban networks that can change basic scaling relations quantitatively. There are many ways in which social groups can condition individual social interactions. Our aim here is to build a simple model that will allow us to modulate the predictions of the previous subsection away from the simple assumption of full social mixing.

**Fig 1:**
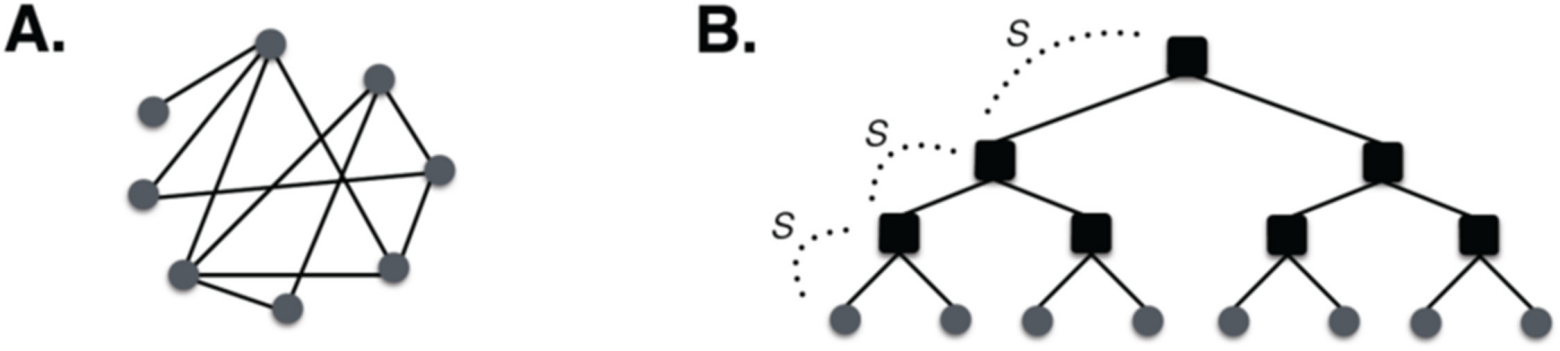
Schematic Social Networks of Towns and Cities. (A) An unstructured network where anyone can in principle connect with anyone else, subject to limitations deriving from cost of movement. Such a network is characterized by increasing connectivity with city population size, with mean degree *k*(*N*) = *k*_0_ *N*^*δ*^, *δ* ∼ 1/6. (B) A structured socioeconomic network. In this case, interactions between individuals are regulated by social groups and institutions (black squares) and may be damped by a factor s<1, for each level of institutions involved. If the parameter s<1, the net effect of institutions is to weaken social possibilities and thus reduce agglomeration effects, taking the exponent of the scaling of area with population for settlements closer to unity.

Consider a simple model for an urban social network where social interactions beyond the household are determined by a hierarchy of “institutions” (Fig 1B, c.f. Fig 1A). Such institutions may be formal, in the sense of guilds and parishes, or they may be more informal such as kinship groups, ethnicity, and social class. Taken together, these different group affiliations may *restrict interaction* between individuals beyond the constraints imposed by distance and transportation costs discussed above.

To quantify the effects of social and economic groups, consider again a settlement with a population of size *N*. We assume that each individual in the settlement belongs primarily to a single group at the lowest level, and that through this group connections may also belong indirectly to other groups at higher levels of the hierarchy. In practice, this means that although people can in principle interact with others simply by moving over space, as in the social reactor model, they will not do so if their group affiliations are incompatible. That is, beyond the local level—the household—we assume that possible social interactions are mediated by these groups only. The hierarchy of groups in Fig 1B is parameterized by *h* levels and at each level we assume that *b* connections are possible. Thus, *b* is the average branching ratio in this hierarchical network. This is similar to an organizational chart for a company, and thus, suggests conceptualizing the city in analogy to a classical firm [84]. The relationship between *N* and *b* is familiar for any balanced tree graph, as *h*(*N*) = log_b_ *N*.

Clearly, even a hierarchy such as that depicted in Fig 1B allows every individual to make contact with any other, in principle, through higher nodes (groups) in this structure. So a final ingredient of this model is that institutions can “dampen” or restrict such interactions. This results in a more divided social network, with institutions as the gatekeepers for contact between individuals. We assume that there is a damping factor, 0 ≤ *s* ≤ 1, that affects each link controlled by a group. This number, like *b*, is in general specific to each relationship, leading to a more complicated but straightforward generalization of the model that we do not pursue here.

The hierarchy depicted in Fig 1B is a simple instance of a more general situation long studied by social scientists; namely, the relationship between social position, power and control [85,86]. We emphasize that this model does not equate to the mere existence of hierarchical institutions, a pervasive situation in both pre-modern and modern societies. Rather, the structured social interaction model supposes that there are fewer social interactions that occur outside of these hierarchical networks. It is also important to note that, if such restrictions would be circumscribed to only negative interactions, such as violence, or if these institutions could *reduce* the cost of interactions by acting as central places or clearing-houses, they could instead contribute to *stronger* agglomeration effects. This can be modeled by considering values of *s*>1. Many modern institutions, such as universities, are built with the intention of promoting positive social interactions, related to innovation and economic growth, beyond what is possible by chance within a city [87].

It is now easy to compute the number of contacts an individual can have as higher-level groups mediate his/her interactions. The crucial parameter is the *social horizon*, *r* = *sb*. If *r*>1 (s>1/b) then the city persists as an integrated socioeconomic system. For r<1 (s<1/b) the social horizon is constraining and the city is increasingly divided into a number of groups dictated by affiliations.

To see this, consider the number of interactions for a typical individual. At the first level, there are *b* interactions, at the second there are *b*+ *sb*, at the third *b*+*sb*+(*sb*)^2^ and so on. The total number of connections of an individual, at a given level of dampening, is then the sum of the finite geometric series

$$k_{s}\left(N\right)=\;b\left[1+sb+{\left(sb\right)}^{2}+\ldots \right]=\;b\frac{1-r^{h}}{1-r}\cdot$$ (3)

For very small *s*, *r* << 1 and *k*_*s*_(*N*) = *b* / (1 − *sb*) ≅ *b*, that is, the interactions stay essentially circumscribed to the household and there is no city at all. Conversely, for *s* close to 1, *r*>1 and we can write

$$k_{s}\left(N\right)=b\frac{{\left(sb\right)}^{h-1}-1}{sb-1}\approx s^{h-1}k\left(N\right)=N^{-\theta }k\left(N\right),\text{ with }\theta =\left|\frac{\text{ln}s}{\text{ln}b}\right|,$$ (4)

where *k*_*s*_(*N*) is the average connectivity per individual in the structured interactions model and where we assumed that the terms that do not involve *s* correspond to the connectivity computed in the previous subsection, $k\left(N\right)=K\left(N\right)/N\sim \frac{N}{A_{n}}\sim N^{\delta }$, since in that case the groups do not exert any effect on connections, which are only limited by effort and spatial and movement costs [55].

Thus, in this limit as *s* → 1, the exponent *θ* vanishes. However, while *θ* is non-zero, it produces a negative correction to standard agglomeration exponents, making them effectively weaker. If we write the productivity of a city as proportional to its connectivity [88,89], and use the expressions from the social reactor model (above), we conclude that, in the case when the restrictive effect of institutions is small but non-zero, the built up area of the city scales as:

$$A_{n}\sim \frac{N}{k\left(N\right)}\sim N^{1-\delta +\theta }\;\cdot$$ (5)

In the opposite limit, when institutions are very restrictive, *A*_*n*_∼*N*, leading to no population densification (*n* = constant) with settlement size. This shows how hierarchical institutions that limit social opportunities weaken and can even destroy socioeconomic agglomeration effects, spatial densification and ultimately cities themselves. Although the structured interactions model is an extension of urban scaling, and reduces to it as the restrictive effects of groups vanish, its strong consequences are measurable if we observe linear scaling of settled area with population in any urban system. While it remains true that such an effect could be due to other forms of segregation, it allows us to at least identify situations where the socioeconomic restrictions from strong group affiliation *could* be at play, an interesting question in the context of medieval European cities.

## Medieval European Urban Systems

### Networked Interactions in Medieval Cities

By the early fourteenth century, the urban systems of Western Europe had undergone centuries of continuous socioeconomic and demographic development. These mature urban networks performed many key political and economic functions for the polities and economies in which they were embedded. While some city dwellers engaged in supplementary food production and other extractive pursuits, by the fourteenth century urban populations were fully engaged in non-agricultural production—and dependent on market exchange to meet their subsistence needs [3–7,14,23,45,47,90–92]. Thus, as nodes of exchange, consumption, and production within wider urban systems, medieval European cities supported interdependent economic networks that facilitated a well-developed division of labor [3–6,11,13–15,18,46,64,93].

Many landed aristocrats, clergy, and wealthy merchants resided in cities and stimulated demand for the provision of luxury goods and services. Elites created networks of market-based patronage, connecting themselves to urban specialists like traders, middlemen, artisans, and skilled laborers to service this demand. Elites often controlled urban craft industries, using urban labor to manufacture goods for export such as textiles, clothing, leather goods, and metals. These sectors in-turn stimulated considerable demand for agricultural produce and other basic goods and services, which were facilitated by market-based transport and construction, skilled craftsmen, and retail middlemen [3–7,10,13–16,18,45,46,90,94]. The strong hierarchical interdependence of these networks both created and required frequent and intense social interaction, but it is unclear how fluid such networks were regarding social and economic mobility.

In addition to economic networks, the hierarchical networks that constituted the urban division of labor were organized into tightly-integrated sociopolitical institutions that controlled the flow of people, goods, and information. Guilds integrated the sociopolitical networks of economically-defined trades, craft industries, merchant communities, and civil governments (burgesses, burghers, bourgeoisie, etc.) to protect their locally-defined common interests. Likewise, Catholic parishes integrated the sociopolitical networks of spatially-defined neighborhoods of households, providing a basal infrastructure for ecclesiastical governance. The social and economic life of most individuals was strongly constrained by the household, which formed the social and spatial context for work and life. Taken together, patronage, households, guilds, and parishes were responsible for the local provision of public goods, thereby extending their reach into all facets of social life [4–7,46,48,49,95,96]. The extent to which these institutions determined the social and economic networks in medieval cities is an important question that we address here via scaling analysis.

### The Impact of Networked Interactions on Spatial Density

Having undergone roughly two centuries of continuous growth, the spatial morphology of mature medieval cities *ca*. 1300 bore the imprint of the processes from which they developed. Because people lived where they worked, urban economies largely determined the spatial patterns of urban demography. In general, commerce and key urban institutions were most concentrated in dense urban nuclei—largely relegating industry trades towards the suburban periphery [1,4–8,97]. Commercial intensification generally led to more dense usage of core urban space. Different socioeconomic groups were often spatially segregated within settlements *ca*.1100, with elites occupying the core areas, and a gradient of different trades radiating towards the periphery. But as cities grew over the following centuries, the growing elite demand for luxury goods and services increasingly led to the diffusion of craft specialists and artisans throughout core urban neighborhoods. Likewise, the expanded urban need for basic goods and services promoted the diffusion of their providers into core areas as well. The resulting modular, vertically-integrated socioeconomic neighborhoods thus became increasingly dense, with the poorest households living in attics, along back alleys, and in the rear of lots. This process was especially prominent in larger cities, political capitals, and mercantile centers swollen with more aristocrats, merchants, and clergy [4–8,46,97–101].

At the same time, other factors promoted spatial diffusion via suburban expansion. Foremost among these was the spatial segregation of economic sectors. Environmentally problematic trades like butchers were often located nearer the periphery. The transport logistics and storage of bulk commodity trade often necessitated a location nearer to peripheral warehouses, harbors, and riverfronts. The powerful occupational guilds of major craft industries (e.g. cloth or fullers) often concentrated in segregated suburban locales in order to inspect and control production and marketing [4–6,8,97–100,102]. While suburbs were home to both rich and poor, they were often home to the shantytowns of recent immigrants and other ‘undesirables.’ Because of the ethnolinguistic diversity of medieval regions, recent immigrants also often settled in segregated cultural neighborhoods near the outskirts [4–6,8,103–107]. These processes of diffusion were more prevalent in larger cities with bigger and more diverse economies [4–8,46,97–106].

Thus, in general, the population density of medieval European settlements was related to the intensity of social interaction—ultimately stemming from the spatial unity of household and occupation. Whereas commercial intensification resulted in dense nuclear agglomeration, the need or desire for social isolation and segregation resulted in diffuse suburbanization.

The two general trends outlined above were systematically mitigated by other factors that imposed spatial limitations. Aside from variation in local topography, the urgency of fortification and the capacity to expand city walls were paramount. Typically enclosed by walls to some extent, the suburban sprawl of fourteenth century cities could sometimes extend beyond the walls for a kilometer or more [4–8,97,98,108–110]. Because they also constrained the flow of trade, some towns built walls well beyond their settled areas, enabling intra-mural suburban sprawl. In contrast, cities whose spatial expansion was restricted by walls were generally much denser, as expanding the walls was expensive and took time. Old walls were rarely torn down—instead providing a framework for the polynuclear internal division of neighborhoods, parishes, and quarters that further circumscribed the growth of core urban space [4–6,8,53,99,109,111].

Incremental growth within these spatial constraints necessitated consecutive morphological changes by increasingly ‘filling in’ unused areas and increasing density. As such, the relative size and age of medieval cities was correlated with population density. Newly established “planned” settlements were laid out to meet particular functions (fortification, church, market) in the absence of demographic pressure. But over time, incremental urban growth often led to the process of urban lot in-filling (known as the “burgage cycle”), suburban expansion, and re-fortification [4–6,52,91,101,110,112,113]. In this way, regional patterns of fortification, and the spatiotemporal magnitude of ‘organic’ urban growth, may be expected to modify the spatial (i.e. population density) outcomes of commercial intensification and suburbanization.

### Regional Urban Systems

**Fig 2:**
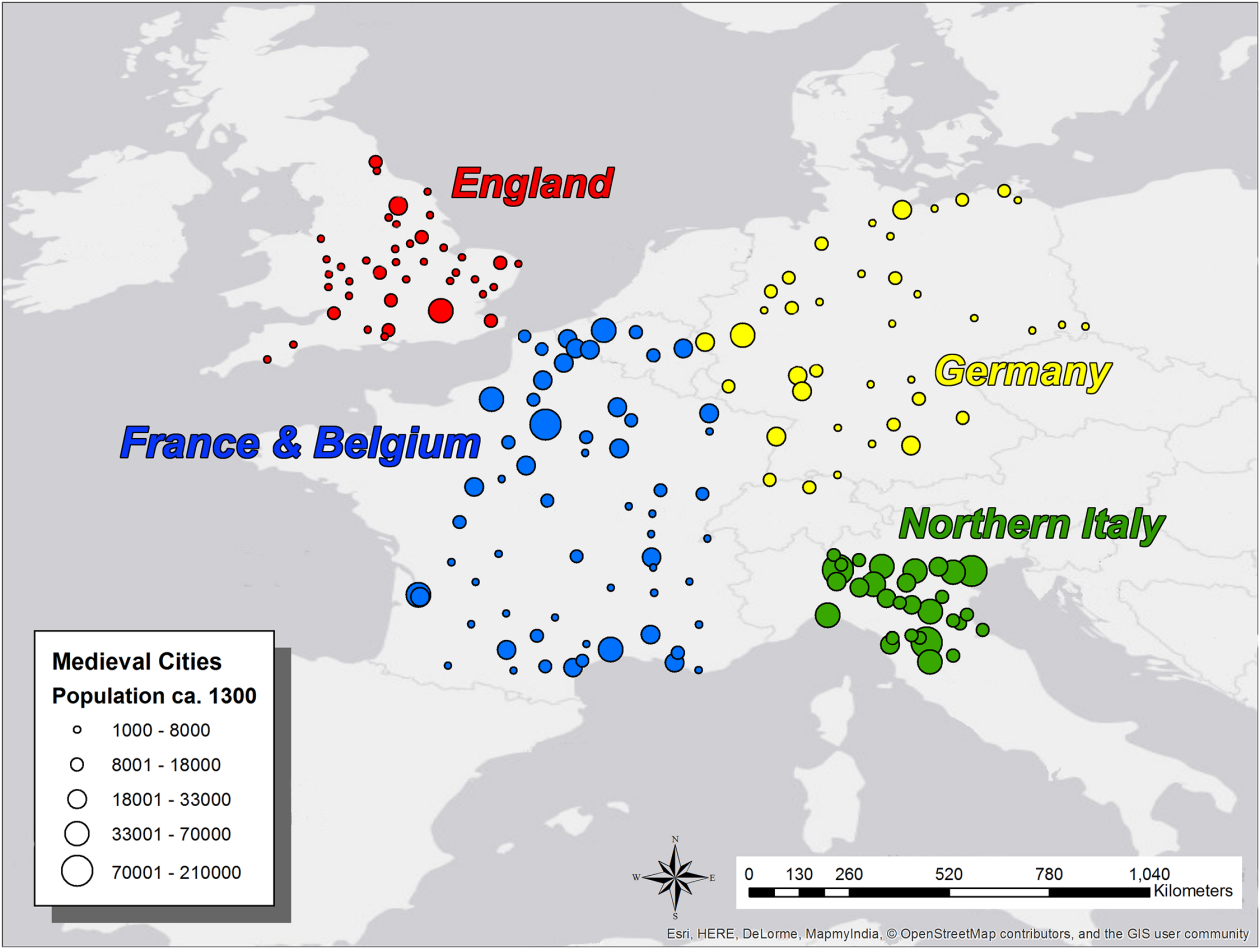
Map of Western European Settlements *ca*. 1300 CE Examined in this Paper. Medieval towns and cities of Western Europe *ca*. AD 1300 examined in this paper (n = 173), in England (red; n = 40), France and Belgium (blue; n = 63), Northern Italy (green; n = 30) and Germany (yellow; n = 40). All settlements examined have populations of >1,000, and in most cases have populations >5,000. This map was created using ArcGIS^®^ software by Esri, © OpenStreetMap and contributors, Creative Commons-Share Alike License (CC-BY-SA).

Historians have noted important differences among the cities of medieval Western European regions. It is important to highlight the differences among regional urban systems because they influenced the character and productivity of intra-urban social networks, their embodiment in urban space, and thus the scaling relations we investigate [64,65,78]. To account for this variation, we divide our database into four regional urban systems in the analyses that follow, in addition to considering the dataset as a whole. Fig 2 shows the locations of the urban system groupings we examine *ca*.1300 (using ArcGIS software and ESRI OpenStreetMap and contributors basemap).

Regional patterning in the factors discussed above may have been most important for structuring intra-urban spatial modes of social network organization. As such, we here define “urban system” as a geographically continuous network of interconnected cities with strong political, economic, and sociocultural ties. The resulting regional groupings used in our analysis are justified and contextualized below. In the S1 Appendix we investigate the effects of historically-plausible groupings using alternative criteria and find that they have little effect on our results. We therefore present our results for the most historically valid groupings in the main text, and interested readers can explore this issue further using the S1 Appendix, S1 Main Data, and S1 Supplemental Data.

#### Northern Italy

The cities of Northern Italy formed a distinctive network of historically tethered and strongly interconnected city states. With a long history of urban growth, Northern Italy was one of the most heavily urbanized regions of Europe by AD 1300 [11,114]; see Fig 2. While nominally part of the Holy Roman Empire, Northern Italian cities’ political and economic fortunes were effectively controlled by rival city-state polities [50,51,115]. Regardless of whether they were directly controlled by landed aristocrats or merchant elites, political and economic power was concentrated in relatively few hands. With this hegemonic power, Italian city-state capitals dominated their political subordinates through redistribution, institutional privileges, and monopoly. This ensured that commodities, capital, and labor flowed into capitals at the expense of minor centers [4–6,14,16,50,51,93,114,116]. The Italian urban network was Europe’s primary hub of international and long-distance trade, a major source of commercial demand for goods and services of all kinds, and the birthplace of sophisticated financial institutions [4,5,12,13,16,93,115]. Italian cities were also major centers of guild-based textile manufacturing and craft production in artisanal suburbs [4,5,11,13–16,93,117,118]. However, because larger Italian cities dominated their smaller neighbors politically and militarily, walls imposed a crucial constraint on the spatial growth of suburbs in most cities [4–6,14,16,99,115,119]. Taken together, the commercial integration, abundance of capital city loci of elite demand, necessity of walls for spatial expansion, and long-term demographic scale of Italian cities *ca*.1300 imply densely compacted socioeconomic networks.

#### Germany

Confederated by the Holy Roman Empire, many urban centers across Germany were granted autonomous status as “Imperial free cities.” Political and economic power strongly overlapped in these cities, and by *ca*.1300 political control was increasingly shifting from landed aristocrats to merchant elites [4–6]. Unlike the Italian city-states, larger cities in Germany dominated regional urban economies through protective trade policies and industrial monopolies (as opposed to direct political and military control). Membership in urban confederations like the Hanseatic and Rhenish Leagues further reinforced the economic prowess of large commercial centers by securing long-distance trade routes, providing shared juridical enforcement, and enforcing mutually beneficial protective trade policies at within the wider urban system [4–6,18]. Expanding along continental and Baltic-North Sea trade routes, major urban centers across Germany developed mature commercial economies, and capital and labor markets that commanded long distance trade flows [4–6,99]. Despite their confederation, however, the cities of Germany remained parts of multiple rival (and sometimes hostile) polities. The combined importance of trade and occasional defense thus made wall expansion around suburbs a priority among German cities, although not to the same degree as in Italy [4–6,120].

#### England

As part of a large and unified territorial state, England’s urban institutions were surprisingly decentralized. The political institutions of English cities were increasingly controlled by the enfranchised merchant elites (burgesses) of independent municipalities by *ca*.1300, and only indirectly connected landed elites through economic ties [15,95,96]. In contrast to the political privileges of Italian city-state capitals, the English urban network developed decentralized market-based interdependencies among both large and small towns [14,15,45,90,91,95,96,121]. As Europe’s largest wholesale exporters of wool and woolen cloth, English urban economies were increasingly stimulated by the development of associated commercial institutions and subsidiary industries. Larger English cities became central nodes in a mature urban economic hierarchy [14,15,43–45,90,91,94,121] in which capital investment and the economic integration of gentry, clergy, and merchants were increasingly incentivized [15,121–124]. This resulted in booming craft industries and commercial specialization [15,45,90,117,121]. Domestic demand for these grew [15,45,90,94,95], and rural migrants poured into growing urban labor markets [14,95,105,106,125]. The combined magnitude of trade, industry, and urban growth translated into extensive urban growth. With few military threats, the suburban sprawl of most English cities remained unenclosed and largely uninhibited [8,97,98,101,102].

#### France and Belgium

As in England, the cities of the vast Capetian French polity and its vassals experienced enormous growth and integration during thirteenth century. The crown enfranchised urban merchant elites (bourgeois) with political freedoms and economic incentives, which stimulated the rapid development of urban craft industries, trade, and commercial specialization to meet the demand of the growing French aristocracy [4,10,46,52]. Capetian vassals also retained formal control over political and economic policies, which played a key role in the success of pivotal urban commercial institutions like the Champagne fairs [10,52]. The gradual Capetian annexation and integration of western border regions extended the French urban network into Burgundy, Dauphiné, Franche-Comté, and Provence. Likewise, the expansion of Capetian political and economic interests into central and southern France saw the rapid integration of these regions into the French urban system via the enfranchisement of existing urban centers and the establishment new, planned towns (*bastides*) [46,52,59,126–131]. Given the backdrop of Capetian military hegemony, cities across France neglected to enclose their rapidly expanding suburban sprawl [4–6,99,110,130]. By the late thirteenth century, the centralizing reforms of Phillip IV—which retracted city self-government charters, centralized urban administration under the aristocracy, and restricted the flow of trade through the Champagne fairs—further cemented the integration of the French urban system [4–6,10,46,52,110].

Like the other neighbors of Capetian France, the cities of modern Belgium, specifically Flanders, Artois, Hainault, Brabant, and Wallonia, were increasingly integrated into the French urban network during the thirteenth century. To be sure, urban self-governance in the Low Countries played a major role in the development of urban capital and labor markets, extensive craft and textile industries, commercial institutions, and long-distance trade [4,18,53,132]. But the Capetian monarchy repeatedly tried to impose vassalage and annexation on both free cities and seigneurial counties across Artois, Hainault, and Flanders from the late twelfth to the early fourteenth centuries. These areas became increasingly dependent on French grain, access to the Champagne fairs, and French demand for urban manufactures. By 1300, French royal currency had replaced local coin in the Low Countries’ economy, and pro-French noble factions formed a major segment of fragmented urban elites. Indeed, the regional economic disruption caused by the reforms of Philip IV and his repeated invasions of, and compromises with Flanders only served to further integrate the region [4,5,10,46,53,126,132]. Because of the need for defense and to control trade, wealthier cities in the Low Countries were often characterized by extensive walled circuits—if they bothered to extend their walls at all—which enabled indefinite suburban growth [4–6,53,110,133].

## Results

### Model Implementation

To evaluate the the data against the predictions of the two models developed above, we compare OLS regression parameters of the log-transformed population and settled area estimates. More specifically, the log-linear OLS estimates for the two regression parameters are directly compared to the corresponding theoretical expectation. The hierarchical interaction model predicts that the scaling exponent *α* will increase (approaching 1, and potentially >5/6) in contexts where social interactions were relatively constrained by hierarchical social institutions. As noted above, the predictions and confidence intervals of the two models overlap, so we can only exclude the structured interactions model in the limit of strong segregation. Although this inhibits statistical hypothesis testing, if hierarchical institutions have a dampening impact on social interaction, we would expect the scaling exponent of the urban system to be systematically closer to 1. As such, we suggest that scaling exponents ≳ 5/6 should be considered candidates for hierarchical institutional dampening effects, and scaling exponents >> 5/6 strong candidates.

**Fig 3:**
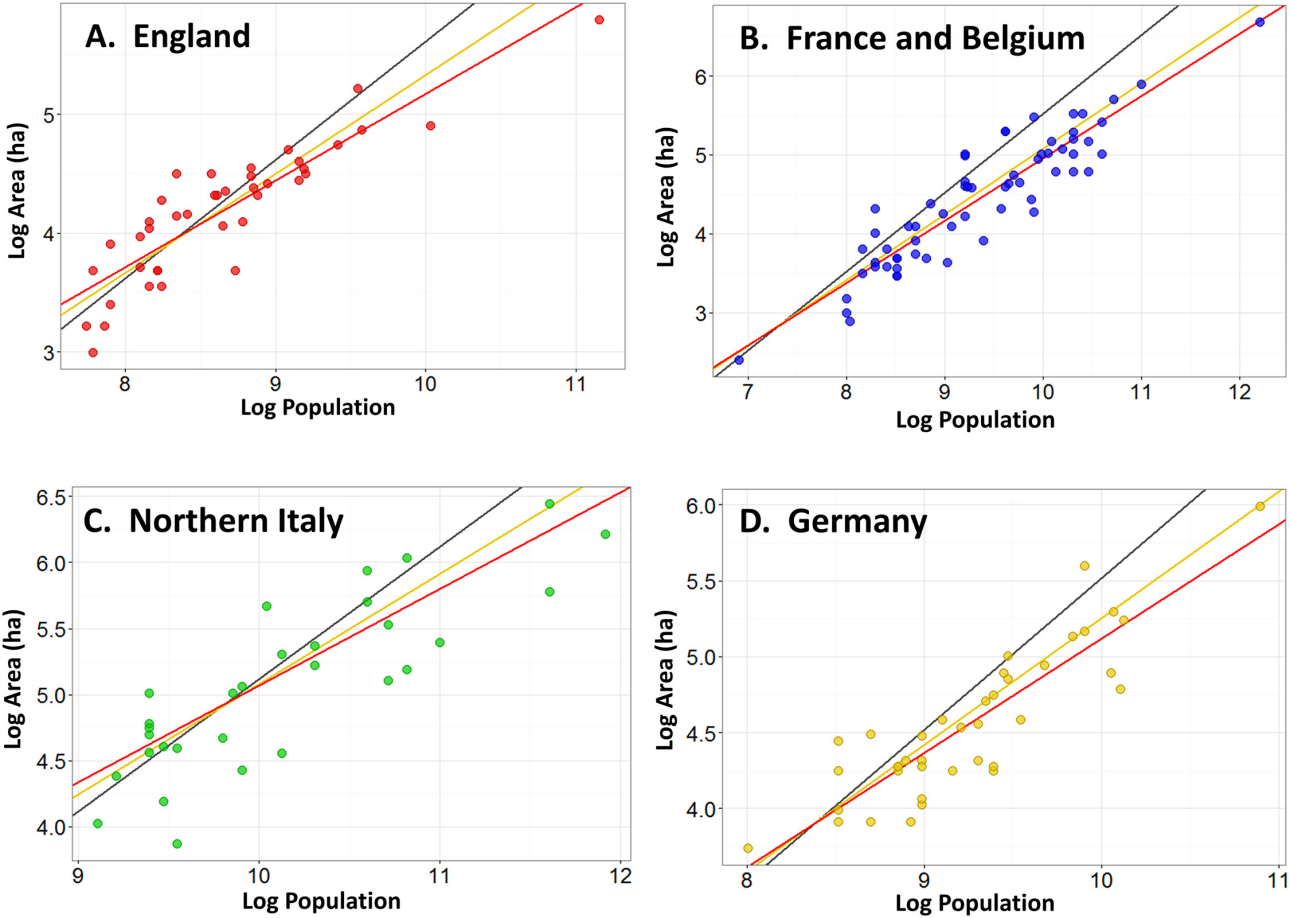
Estimation of Area—Population Scaling Relations for Regional Urban Systems. Estimation of Area—Population scaling relations for: (A) England (red); (B) France and Belgium (blue); (C) Northern and Central Italy (green); and (D) Germany (yellow). The black line represents proportionate (linear) scaling; the yellow line the theoretical prediction where *α* = 5/6; and the red line the best-fit line from OLS regression of the log-transformed data.

**Fig 4:**
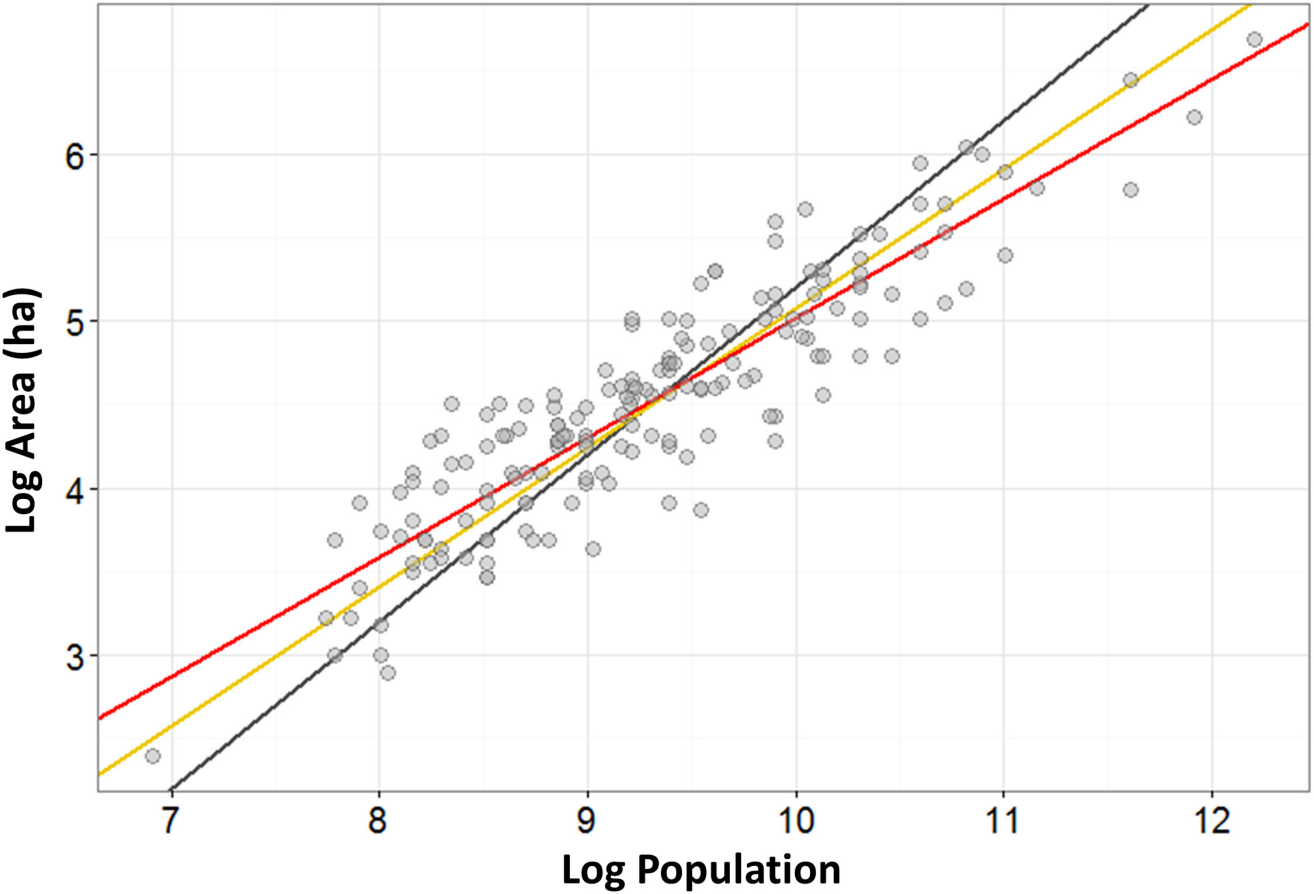
Estimation of Area—Population Scaling Relation for All Settlements. The Area—Population scaling relation for the entire data set of all medieval cities (*n = 173*). The black line represents proportionate (linear) scaling; the yellow line the theoretical prediction where *α* = 5/6; and the red line the best-fit line from OLS regression of the log-transformed data.

We estimate scaling exponents and prefactors through ordinary least-squares (OLS) regression of the natural logarithm of areal extent against the natural logarithm of population size:

$$\text{ln}\left(area_{i}\right)=\alpha +\beta \text{ln}\left(population_{i}\right)+\xi$$ (6)

where *i* indexes a city within a specified urban system and *ε* denotes an i.i.d. Gaussian white noise. Eq (6) was estimated using OLS with the Huber/White correction for heteroscedasticity. Estimations were done using R version 3.2.2 software and the “sandwich” heteroscedasticity-consistent covariance matrix package [134,135]. Scatter plots for the dependent versus independent variables show linear relationships indicating that the model is not misspecified for either the regional urban systems (Fig 3) or the pooled data (Fig 4). The regression results for four regional European urban settlement systems, and the pooled dataset, are given in Table 1. Note that eq (6) is merely the log-transformed version of eq (1), and thus *β*, the scaling coefficient in the log-transformed case, is also the scaling exponent α in eq (1). In the same way, *β*_*0*_, the intercept in eq (6), is related to the prefactor *a* in eq (1) by $a=e^{\beta_{0}}$.

**Table 1:** Scaling of Settlement Area with Population Size: *ln(area) = α + βln(population)*.

Model	*N*	*adj-R*^*2*^	*α (S*.*E*.*)*	*α C*.*I*.	*Β (S*.*E*.*)*	*β C*.*I*.
France & Belgium	63	0.84	-2.942 (0.334)	[-4.045, -1.839]	0.790 (0.035)	[0.665, 0.914]
England	40	0.79	-2.124 (0.551)	[-3.240, -1.008]	0.730 (0.062)	[0.604, 0.856]
Germany	40	0.77	-2.422 (0.623)	[-3.681, -1.163]	0.754 (0.068)	[0.616, 0.891]
N. Italy	30	0.71	-2.23 (0.764)	[-3.795, -0.664]	0.720 (0.075)	[0.566, 0.874]
All Cities	173	0.81	-2.125 (0.248)	[-2.615, -1.635]	0.714 (0.026)	[0.662, 0.766]

**Notes:** Estimations done using OLS with corrections made for heteroscedasticity. Standard errors are in parentheses, and confidence intervals are in brackets (all scaling coefficients are sig. at the .05 level). See text for details.

### Analysis

Table 1 shows that the point estimates of the scaling coefficients for all four regional groups and for the pooled dataset fall within the 2/3 ≤ *α* ≤ 5/6 range predicted by the social reactor model. The 95% confidence intervals for these exponents exclude 1 in all cases. Thus, medieval cities across Western Europe exhibit, on average, economies of scale with respect to spatial agglomeration such that larger cities were denser on average. This pattern is similar to that observed for modern cities, although the medieval values are somewhat closer to 2/3, whereas in modern cities they are typically closer to 5/6.

It is notable that the estimated prefactors cluster quite closely together, with point estimates for the prefactors varying only between 0.05 and 0.12. According to the models above, *a* should vary in accordance with differences in the relationship of interaction benefits to intra-settlement transportation costs across urban systems. Since transportation technology was relatively constant across medieval Europe, any differences in the scaling prefactor should reflect differences in the productivity of social interaction. In general, this suggests that average net benefits of social interaction were relatively constant across Western Europe *ca*.1300.

More importantly, the scaling coefficients are quite similar across regional urban systems (and ithe pooled data), with point estimates varying between 0.71 and 0.79. All of these are within the confidence range (2/3 ≤ *α* ≤ 5/6) predicted by the social reactor model, and none are ≳ 5/6. For this reason we cannot detect evidence for socioeconomic interaction-dampening caused by hierarchical institutions. Such institutions may still have caused a degree of dampening, as the two models’ predictions are equifinal within the range 2/3 ≤ *α* ≤ 5/6. Nevertheless, the consistent values of the estimated scaling exponents imply that average net socioeconomic interaction benefits fell within the modern range and were not dampened towards unity. This suggests the hierarchical institutions of medieval urban systems did not have a strongly-restrictive impact on urban socioeconomic interactions, at least along the lines predicted by the structured interactions model in the aggregate population-area relationship of these settlements. In the S1 Appendix we demonstrate that variation in both estimated regression parameters is so low across groups that any differences between them are not statistically significant (S1.3). For this reason, we refrain from interpreting the differences in estimated parameter values among urban systems.

## Discussion

Despite their many structural differences, and a temporal distance of 700 years, medieval urban centers share at least one basic similarity with modern cities: larger settlements have higher population densities than their smaller counterparts within a given urban system. Overall, the data conform to the expectations of the social reactor model, as the population-area relation does not provide evidence for disappearing scaling effects. Even though medieval cities were structured by hierarchical institutions that are ostensibly not so dominant today, we interpret this finding as excluding a strongly segregating role for medieval social institutions. This would suggest that the institutions of Western European urban systems *ca*.1300 did not substantially constrain social mixing, economic integration, or the free flow of people, ideas, and information. We take these findings as an indication that the underlying micro-level social dynamics of medieval cities were fundamentally similar to those of contemporary cities. Notwithstanding their many structural and functional differences, and contrasting macro-level processes that influenced urbanization in each regional system, both medieval and modern cities appear to be characterized by social networks that become increasingly spatially-dense as they grow.

The results presented here also have contextual implications for understanding urbanization in Western Europe *ca*.1300. In particular, the prevalence of strong spatial agglomeration across fairly diverse urban systems implies that inter-regional differences were limited in their consequences. As noted above, Italian cities might have been expected to exhibit stronger densification with city size due to numerous capital cities, many centuries of organic urban growth, and circumscription by defensive walls. But the observation of statistically indistinguishable agglomeration effects in England, France and Belgium—with uninhibited suburbs, fewer political capitals, and a much larger proportion of relatively young cities—suggests that the common process of commercial integration and concomitant demographic nucleation was the primary driver of this trend. Even though larger cities had greater impulses toward suburbanization, larger cities were nevertheless increasingly dense. As such, the forces that caused agglomeration outweighed those causing suburbanization and segregation. Based on the assumption that the observed demographic-spatial agglomeration was driven by socioeconomic network effects, it might therefore be argued that the net impact of medieval urban institutions was to increase per capita interaction rates in order to facilitate greater organizational efficiency, productivity, and functional diversity.

This emphasis on intra-urban interaction in no way negates the influence of macro-level urban system dynamics on the historical evolution of those urban systems. Rather, we argue that urban system dynamics emerge precisely because of the capacity of cities to facilitate greater social interaction and productivity [78]. Indeed, medieval European urban systems were central to the development of socioeconomic and political institutions that expanded the division and coordination of labor, centralized networks of commodity flows, and intensified political and economic organization [1,3–6,9–18,24,27]. These systemic processes are well documented in historical sources, and our analysis offers a quantitative model for the intra-urban causes of their emergence.

The similar quantitative relationship between areal extent and population size exhibited by medieval European urban systems and contemporary urban systems also suggests that modern and medieval cities may share similar underlying social processes. In this way, theories of contemporary urban processes (e.g. from urban economics or economic geography) may be applicable to the past not because of similarities in macro-historical structures (e.g. political and economic institutions), but because of fundamental similarities in micro-level behaviors (e.g. agglomeration and interaction networks) and their emergent system-level outcomes (urban land use, division of labor, and economic productivity in cities). That medieval and contemporary cities might be united by a common set of underlying social processes is not to say, of course, that they shared all such processes, or that there weren’t social processes operative in the past which do not apply to modern urban life. But rather than assuming that modern theories either do or do not apply to the past, this approach forces us to consider *how* and *why* past social, economic, and political conditions impacted the structure and dynamics urban systems.

By providing an analytical framework and a set of theoretical expectations, scaling analysis can make contributions to a wide variety of topics in social and economic history. Indeed, settlement scaling theory operates in terms of quantities that are common to human settlements regardless of time and place, similar to rank-size analysis and the methods of central place theory. With larger and more comprehensive datasets it may be possible to compare scaling relations across smaller urban regions, which may shed light on important differences among them. Time-series data on the changing scaling relations of urban systems may also reveal crucial long-term patterns in their evolution. Likewise, scaling analysis of large, quantitative urban morphology datasets might help assess general patterns in the intra-settlement dynamics of the built environment. If such analyses are successful in identifying patterned variation associated with specific historical contexts, the theoretical framework of settlement scaling will be able to produce useful historical contributions to the study of pre-modern urbanization. It is our hope that scholars of history will engage with, cross-pollenate, and critique such research as we carry it into the future.

## Materials and Methods

Although our data represent only a sample of the settlements known to have been inhabited during the early fourteenth century, we refer to these data as collectively characterizing “medieval Europe”. Similarly, we use the term “city” to describe settlements that medieval scholars have often referred to as “towns,” even though the majority of our cases had between 2,000 and 10,000 inhabitants. Settlements with fewer than 10,000 people were of crucial importance to medieval European urban systems, and our inclusion of them in a database of “cities” is consistent with our aim of investigating how settlement space responds to population at multiple scales.

The database was constructed by collecting population and settled area data on medieval settlements in Western and Central Europe from both the secondary literature and tertiary databases. By “secondary sources” we mean historical and/or archaeological analyses of medieval cities, and by “tertiary databases” we mean scholarly compilations of urban data (e.g. Bairoch et al., 1988). No primary historical or archaeological research was conducted in the construction of the database. Settled area estimates were either measured from published historical and archaeological maps (using ImageJ software), or from scholarly estimates in the secondary literature. Population estimates were drawn from the secondary literature and tertiary databases. Each “case” is a settlement, comprising both population and area data *derived from independent evidence*; we did not use population estimates based on settled area extrapolations. All population and area estimates were focused on the early fourteenth century, roughly spanning a 40 year period from *ca*.1280-1320. All cases had populations of greater than 1,000, and most cases had populations >5,000. Our dataset includes 173 cities located in modern-day England, France, Belgium, Germany, Switzerland, and Italy. These were divided into four urban systems: England (n = 40), central and northern Italy (n = 30), France and Belgium (n = 63), and Germany (n = 40); see Fig 2.

Quantitative data on medieval settlements are inherently contingent on philological interpretations of both population proxies, their geographical referents, secondary reconstructions, and constituent historiographical paradigms. Moreover, the sources to which we had access were of variable quality and abundance across regions. In order to deal with these problems, we developed a data collection protocol that systematically compared and contextualized different quantitative estimates when possible. Rather than blindly assembling data through uncritical use of available sources, our method enabled us to evaluate the degree of confidence in each estimate. The core of this method involved the following steps:

1. We noted the major trends (how were these identified?) in the urban historiography (formal historical methods and assumptions) of medieval regions from the secondary literature;
2. We collected population and area estimates from works representing the most recent school of thought about each place, whenever possible;
3. We noted population and area estimates from major tertiary compilations of population and area data [58,59,136]; and
4. We compared the available evidence for each case to arrive at a single provisional estimate suitable for scaling analysis.

### Population Estimation

Conflicting population estimates of medieval settlements are common, and this makes careful source criticism especially important. In cases where recent secondary sources were available, we analyzed the context, methods and reasoning behind each population figure in order to inform our estimate. This was possible for all English cases because the evidence exists on a national scale (nationwide tax rolls), as well as many cases in France, Belgium and Italy. Secondary literature was available for only the largest German cases. Although population data are available from many sources for both England and Italy, we decided to use the well-researched datasets of Campbell [44] and Malanima [114,137], respectively, because of their high quality. These datasets matched our own independent estimates very closely for both regions (see S2 Appendix for more details).

For individual settlements where secondary treatments were scarce or unavailable, we assembled all of the estimates and selected among divergent/contradictory data through internal coherence and crude historiographical criticism. This involved giving preference to estimates made by recent and reliable secondary sources that were written by experts on a particular urban system (e.g. Flanders). The reliability of sources was gauged by how many well-cited research publications an author had on the particular subject and whether the cited reference was an in-depth analysis or a passing comment based on preliminary evidence or assumptions. If scholarly discussions were unavailable, we began to narrow down the set of possible estimates by removing estimates from older sources that stood apart from more recent sources. In the case of tertiary sources, source criticism was based on the estimate’s citation—not the tertiary source alone. We chose single estimates that seemed most accurate given the historical context. If multiple estimates could not be differentiated based on historical context, we took the mode or the average of all available estimates as our point estimate for the population of a given city *ca*. AD 1300 (see S2 Appendix for more details).

### Settled Area

The same procedure was followed for the estimation of settled areas when conflicting estimates were encountered. Topographical map reconstructions of medieval European cities produced by archaeologists and historians are often scattered or embedded in case-specific studies of particular locales—making the compilation and measurement of settled area data a very time consuming endeavor. While it would have been relatively simple to only measure the walled (or fortified) areas of medieval towns, many settlements had shifting extramural suburbs that extended beyond their walls, and others never filled their walled areas completely. Either way, these intramural and extramural areas are reflected in contemporary population counts [59,138–140], which necessitated that our settled area estimates explicitly take extramural suburbs and intramural vacant space into account. Accordingly, our method involved 1) conducting a survey of the archaeological and historiographical literature on medieval urban regions, 2) collecting maps, area estimates, and contextual specifications on each case, and 3) comparing the available evidence of each case to arrive at a single provisional estimate suitable for analysis.

**Fig 5:**
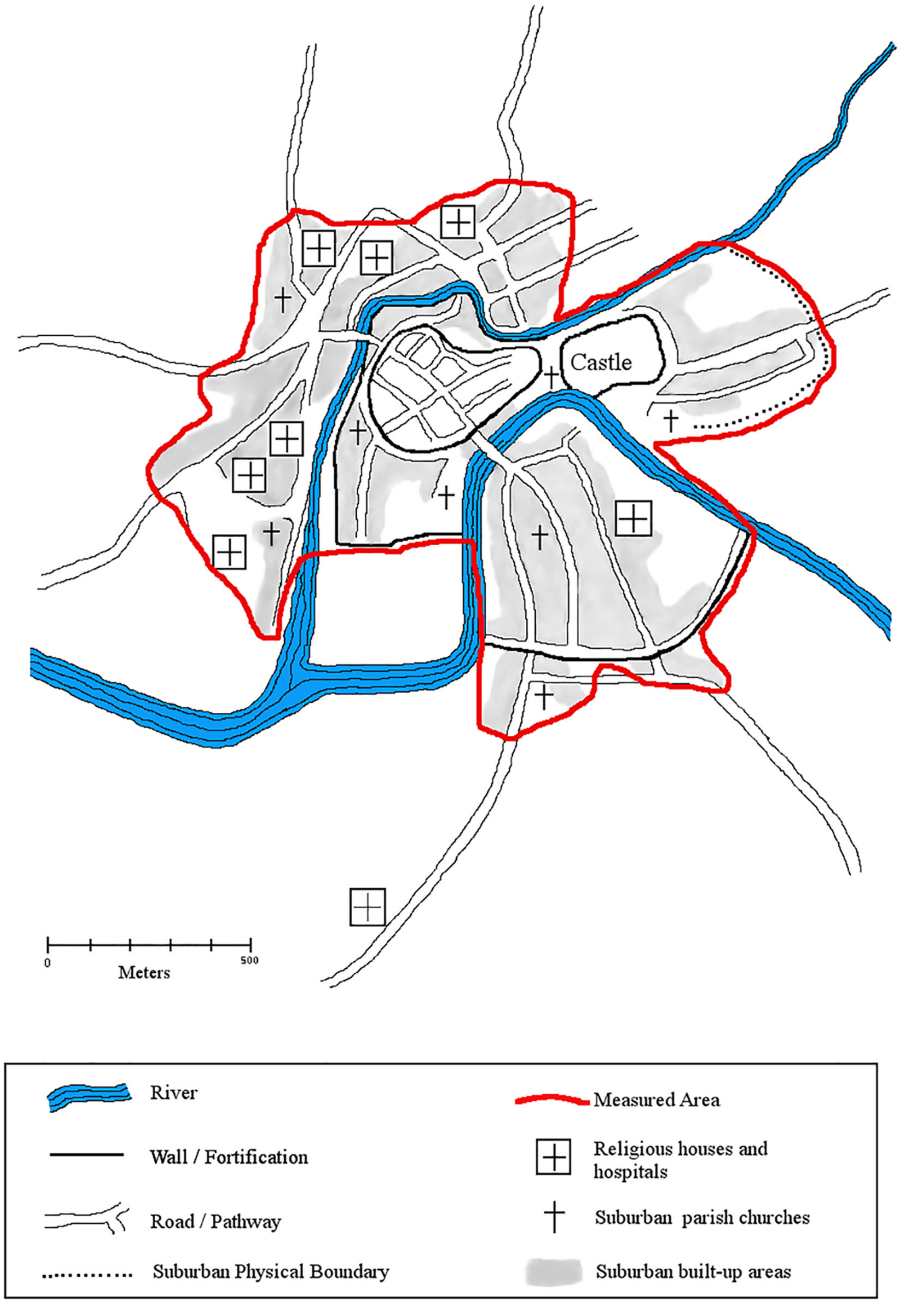
Measuring the Settled Area of Medieval Settlements. Bristol’s built-up areas in the later middle ages (late 13th–early 14th centuries), including built-up suburban areas shaded in grey. The red line indicates the 130 ha settled area we measured for the city, whereas the inner area circumscribed by walls and rivers measures only 55 ha. Even our relatively conservative outline of the city’s built up area more than doubles Bristol’s settled area. This map is modified and redrawn by the authors from Derek Keene’s (1976) map of the suburban built up area of later medieval Bristol [102].

When measuring maps for their settled areas, it was necessary to contextualize the map to understand the chronology of its features and weigh alternative area estimates. For example, we found a number of conflicting area estimates for Bristol *ca*.1300. Bristol’s outer wall was constructed first during the late twelfth century, extended southward during the thirteenth century, and *murage* grants were issued contiguously from 1232 through the fifteenth century [109,141]. Kermode indicates that its walled area was 55 ha [142], but Russell estimated that the settled area had to have been over 80 ha due to suburban sprawl *ca*. 1300 [59]. Most maps of Bristol’s walled area *ca*.1300 match Kermode’s 55 ha [97,111], but leave out their extramural suburban areas. Nevertheless, Keene’s map of Bristol *ca*.1300 (see Fig 5) duly indicates a walled area of 55ha, and a total settled area of 130ha when suburban areas are included [102]. Fig 5 is modified by the authors from Keene´s map of Bristol [102], and illustrates the settled area of Bristol *ca*.1300 (with the measured area outlined in red). This 130 ha is further corroborated by Russell’s earlier suspicion that Bristol must have encompassed more than 80 ha *ca*. 1300. We therefore used the estimate of 130 ha derived from Keene’s map in our analysis.

In other cases we had less information. For example, we collected two area estimates for Carcassone: 68 ha and 40 ha, without a map or detailed context to choose between them. Nevertheless, extramural suburban sprawl is known to have been a very common pattern of cities in the French Midi *ca*.1300 [4,129], so we chose the larger estimate. These examples embody the general process of estimation for each of our cases (see S2 Appendix for more details).

### Assessment of Data Accuracy

Spatially, medieval European cities were nucleated urban agglomerations that were qualitatively distinct from their rural hinterlands [4–7,97,98,108–110]. But while the urban-rural transition was often gradual, these settlements possessed definable spatial boundaries that differentiated them from associated rural hinterlands [6,8]–thus making it possible to measure the areal extent of urban spaces. Because of their spatial, political, economic, and social differences from the countryside, medieval European cities can also be said to have had definite resident populations. Indeed, medieval writers recorded numbers of hearths, soldiers, taxpayers and citizens from distinct urban areas, and it is by extrapolating these figures that historians have estimated their total populations [59,143–147]. These urban populations could fluctuate over time, and the empirical bases for these estimates vary in accuracy and precision across cities.

Despite careful data collection, the match between settled area estimates and population estimates is not always precise. The walls of European towns were increasingly expanded over the course of the later middle ages, and it is well attested that the suburbs of medieval settlements fluctuated greatly, rapidly and asynchronously in accordance with economic and demographic trends [4,5,7,97,98,108,109]. We deliberately collected estimates pertaining to the turn of the fourteenth century (*ca*.1300) because of the relative abundance and quality of the data for this period. Fortunately, our focus here is not on individual estimates, but on the overall relationship between population and settled area within and between regions. Thus, so long as errors in existing estimates are unstructured they should be adequate for the purposes of this paper. Not unlike settled area measurements from nineteenth- and early twentieth-century sources, this level of resolution is simply the reality of the available evidence.

## Supporting Information

S1 Appendix Alternative Urban System Territories. This appendix includes alternative delineations of medieval urban systems, the scaling analysis of these alternative urban systems, and ANOVA for all urban system groupings analyzed in the main text and in the S1 Appendix.
(PDF)

S2 Appendix Database Construction. This appendix includes tables of all historical population and settled area estimates that went into the database, the methods and reasoning behind each data point, a brief overview of how to read these tables, and a comprehensive bibliography of the sources.
(PDF)

S1 Main Data Medieval Cities Data. This is a.csv file of all data analyzed in this paper, including all cities’ population and area estimates (n = 173), as well as their urban system groupings and georeferenced lat-long coordinates.
(CSV)

S1 Supplemental Data Alternative Urban Systems Data. This.xlsx spreadsheet contains the alternative urban system groupings analyzed in the S1 Appendix.
(XLSX)
