# Supplementary material for: Population-Area Relationship for Medieval European Cities
Source: PLoS One. 2016 Oct 5;11(10):e0162678. doi: 10.1371/journal.pone.0162678 (PMC5051806; doi:10.1371/journal.pone.0162678)
Supplement: S1 Appendix — This appendix includes alternative delineations of medieval urban systems, the scaling analysis of these alternative urban systems, and ANOVA for all urban system groupings analyzed in the main text and in the S1 Appendix. (PDF) [file pone.0162678.s001.pdf]

S1 Appendix: Alternative Urban System Territories

S1.1 Overview 1
S1.2 Alternative Urban System Delineations 2
S1.3 ANOVA of Estimated Scaling Parameters 5
S1.4 Bibliography 7

S1.1 Overview

As noted in the "Regional Urban Systems" section of the main text, we define "urban system" as a geographically continuous network of interconnected cities with strong political, economic, and sociocultural ties. We expect that the socioeconomic and demographic patterns of regional urban systems were the most important for structuring intra-urban spatial modes of social network organization. This loose definition is also favored because the different dimensions of evolving urban networks (political, economic, cultural, etc.) have fuzzy, fluctuating territories that never perfectly overlap (*1*, *2*). This flexible definition enables cities like Milan and Genoa—with strong social, cultural, economic, and geographic interconnections—to share an urban system even if they were politically distinct. Likewise, cities that were strongly integrated by state political economies—like Carcassone and Rouen, or Oxford and Scarborough—to share an urban system even if they were economically and culturally distinct. Because the various dimensions of evolving urban networks (political, economic, cultural, etc.) were imperfectly correlated in medieval times, defining functional urban systems in a consistent way is difficult across contexts.

Modern urban systems are predominantly defined in terms of their economic interdependence, and the degree of urban economic interdependence is strongly correlated with city size (*1*). This is especially true for medieval Europe, as pre-modern urban systems were more strongly determined by the capacity of political structures and socioeconomic benefits to outweigh the elevated costs of pre-modern transportation (*2-4*). As such, defining medieval European urban systems purely in terms of economic interdependence would result in an inter-urban network of large, more distant cities like Florence and London instead of smaller, nearby cities like Bordeaux and Périgueux that were regionally intertwined. Indeed, we expect that regional socioeconomic and demographic patterns are the most important for structuring the intra-urban modes of spatial organization. By explicitly defining urban systems in terms of social, political, and geographical relationships alongside economic ones, our definition of urban system enables crucial historical relationships to structure our dataset. Indeed, it should be noted that there is a natural spatial clustering of the data that corresponds to our chosen urban system delineations (see Fig 2 in main text).

In addition to contextual reasoning, our choice of urban systems was guided by the necessity of sufficiently large sample sizes for scaling analysis. Our database must be understood as a sample, as each of our urban regions excludes a considerable number of known cities *ca.*1300 for which settled area estimates were unavailable. Because OLS relies on the central limit theorem to estimate model parameters, parameter estimates from samples where $n \lesssim 30$ cannot be expected to accurately approximate population parameters. For this reason, we refrained from analyzing

urban systems with samples where $n < 30$. As our database expands, larger future datasets will be able to explore smaller urban regions with greater confidence.

Nevertheless, given the imperfect and somewhat arbitrary nature of delineating dynamic and overlapping urban systems, we have supplemented the four urban regions analyzed in the text with alternative, historically plausible urban system delineations that in-turn emphasize political, and regional socioeconomic relationships. These alternative urban system groupings are presented, analyzed, and discussed in S1.2, below. In addition, we have provided the reader with the data necessary to analyze additional urban system delineations (see S1_Medieval_Cities_Data.csv and S1_Alternative_Urban_Sytems.xlsx).

Finally, in section S1.3 we perform ANOVA on all urban system delineations, which is followed by a discussion of the results and general implications.

S1.2 Alternative Urban System Delineations

In order to cross-check the validity of our results, we delineated two sets of urban system territories. While both of these meet the same "urban system" definition outlined in S1.1, above, one set gives strong emphasis to political relationships, while the other set gives strong emphasis to socioeconomic, geographical, and/or cultural relationships. Hereafter we refer to these two alternative groupings as the "political" and "socioeconomic" urban system territories, respectively, for ease of parlance (—although it must be noted that this is not meant to suggest that all other analyzed urban systems do not also share these qualities as well). The exact list of cities included in each of the following datasets is included in the supplemental materials.

Alternative Political Urban Systems

We delineated three primary "political" urban systems based on overarching political allegiances c.1300. Smaller political units were not feasible with our dataset because the sample sizes become too small. These three political units include the following regions in our dataset:

1. The Plantagenet Kingdom, made up of England and the Plantagenet parts of Gascony in the Duchy of Aquitaine
2. The Capetian Kingdom and its vassals, made up Flanders and much of France (excluding Alsace, Lorraine, and parts of eastern Burgundy, Franche-Comte, Savoy, eastern Provence, and English Gascony)
3. The Holy Roman Empire, including Germany, Switzerland, Wallonia, Brabant, Northern Italy, Alsace, Lorraine, and parts of eastern Burgundy and eastern Provence.

In addition to these three overarching political urban systems, we divided the large and heterogeneous Holy Roman Empire into three subcategories that reflect Northern Italy's low level of integration into the Holy Roman Empire c.1300. These include:

i. The entire Holy Roman Empire
ii. The Holy Roman Empire excluding the independent maritime republics of Genova and Venezia

iii. The Holy Roman Empire excluding all of Northern Italy, with Northern Italy as its own political region. (The delineation of Northern Italy here was the same as the original grouping from the text.)

The idea behind this further subdivision was to explore multiple alternative political scaling relationships for the Holy Roman Empire to see if any of them impacted scaling analyses. Scaling analysis was performed on all of the above political groupings.

Alternative Socioeconomic Urban Systems

The "regional socioeconomic" urban systems were delineated to emphasize cultural, socioeconomic, and geographical relationships among medieval cities c.1300. Again, smaller socioeconomic units were not feasible with our dataset because the sample sizes become too small, and some of the regions were very similar or identical to those presented in the text. Several of these urban systems overlap one another as well, so not all of the regions are mutually exclusive (there are a number that can form mutually exclusive sets, and these have been explored in S1.3, below). The ten (n=10) socioeconomic groupings are as follows:

1. Northern Italy (same as in text), with the southernmost regions being Toscana and Emilia Romagna (excluding Umbria, Marche, and Lazio)
2. England (same as in text), excluding Wales and Scotland
3. Central England, excluding Devon, Cornwall, and the counties north of the Humber
4. Northern France and Low Countries, including all of Belgium, Aachen and Trier, and central France; but excluding, Alsace, Lorraine, Burgundy/Franche-Comte/Dauphine, Gascony/Aquitaine, Languedoc, and Provence
5. Northern and Central France, including Alsace, Lorraine, and Burgundy/Franche-Comte/Dauphine; but excluding the Low Countries and Calais, Gascony/Aquitaine, Languedoc, and Provence
6. Southern France, including Burgundy/Franche-Comte/Dauphine, Gascony/Aquitaine, Languedoc, and Provence
7. Mediterranean Europe, including Languedoc, Provence, and Northern Italy
8. Germany, including northern Switzerland, and excluding Aachen and Trier next to Belgium
9. Central Continent, including Burgundy/Franche-Comte/Dauphine, Switzerland, Alsace, Lorraine, Swabia, Franconia, and Bavaria
10. North Continent, including Belgium, Calais and Amiens, Aachen and Trier, and the Hanseatic member cities of Northern Germany

Our aim in constructing these delineations was less towards ironclad historical justification, and more towards assembling a large number of plausible urban regions that fit our urban system criteria (explicated above). This enabled us to grasp the full range of possible estimated scaling parameters from the data. While we acknowledge that smaller urban regions (e.g. Swabia, the Low Countries, Burgundy, Hanseatic cities, etc.) would be more advantageous, we simply do not have a large enough dataset to explore such small-scale urban regions. We intend to explore such scaling analysis in future research.

Analysis

As in Section 4.2, we estimated scaling exponents and prefactors through linear regression of the natural logarithm of areal extent against the natural logarithm of population size:

$$\ln(areal\ extension_i) = \beta_0 + \beta \ln(population_i) + \varepsilon, \qquad (1)$$

where *i* indexes a city within a specified urban system and ε denotes an i.i.d. Gaussian white noise. Equation (1) was estimated using OLS with the Huber/White correction for heteroscedasticity. Estimations were done using R version 3.2.2 software and the "sandwich" heteroscedasticity-consistent covariance matrix package (*5*, *6*). The regression results for the original four regional European urban settlement systems, and the pooled dataset, are given in Table S1.1. The results for the alternative political urban systems (n=6) are shown in Table S1.2, and the alternative socioeconomic urban systems (n=10) are presented in Table S1.3.

Table S1.1. Urban System Territories Analyzed in the Text

Urban System Territories	N	R^2	Scaling Coefficient	Scaling Coef. 95% C.I.	Intercept	Intercept 95% C.I.
**England**	40	0.7899	0.730	[0.604, 0.856]	-2.124	[-3.24, -1.01]
**France and Belgium**	63	0.8404	0.790	[0.665, 0.914]	-2.942	[-4.04, -1.84]
**Northern Italy**	30	0.7135	0.720	[0.566, 0.874]	-2.230	[-3.80, -0.66]
**Germany**	40	0.7732	0.754	[0.616, 0.891]	-2.422	[-3.68, -1.16]
**Europe**	173	0.8101	0.714	[0.662, 0.766]	-2.125	[-2.62, -1.64]

Table S1.2. Regression Results for Political Urban Systems

Political Urban System Territories	N	R^2	Scaling Coefficient	Scaling Coef. 95% C.I.	Intercept	Intercept 95% C.I.
**Capetian France**	49	0.826	0.784	[0.694, 0.875]	-2.893	[-3.77, -2.01]
**Plantagenet England**	43	0.805	0.726	[0.618, 0.835]	-2.107	[-3.08, -1.13]
**Holy Roman Empire (maximum extent)**	75	0.820	0.721	[0.635, 0.808]	-2.197	[-3.02, -1.38]
**Holy Roman Empire (without Genoa or Venice)**	73	0.819	0.749	[0.661, 0.837]	-2.452	[-3.28, -1.62]
**Holy Roman Empire (without Italy)**	51	0.801	0.806	[0.684, 0.928]	-2.949	[-4.06, -1.84]
**Northern Italy**	30	0.7135	0.720	[0.566, 0.874]	-2.230	[-3.80, -0.66]

The alternative political urban systems exhibit the same general sublinear scaling patterns as the original urban systems analyzed in the text, and in no cases did an estimated scaling parameter fall outside the predicted range of the social reactor model $(2/3 \leq \alpha \leq 5/6)$. Indeed, all combinations of political urban systems yielded scaling exponents very close to their closest geographical analogue in the other dataset (< 0.05). The range of estimated scaling coefficients from the original groupings is [0.72, 0.79], and the range for the political groupings is [0.72, 0.80]. Even simple visual comparison of Tables S1.1. and S1.2 shows striking similarities between the estimated scaling parameters and confidence intervals of the two sets.

Table S1.3. Regression Results for Socioeconomic Urban Systems

Socioeconomic Urban System Territories	N	R^2	Scaling Coefficient	Scaling Coef. 95% C.I.	Intercept	Intercept 95% C.I.
**Northern Italy**	30	0.714	0.720	[0.566, 0.874]	-2.230	[-3.80, -0.66]
**England**	40	0.790	0.730	[0.604, 0.856]	-2.124	[-3.24, -1.01]
**Central England**	31	0.800	0.750	[0.58, 0.91]	-2.276	[-3.73, -0.81]
**N. France and Low Countries**	31	0.805	0.786	[0.680, 0.892]	-2.839	[-3.93, -1.75]
**Northern and Central France**	30	0.887	0.843	[0.77, 0.92]	-3.430	[-4.15, -2.71]
**Southern France**	34	0.792	0.727	[0.587, 0.867]	-2.423	[-3.70, -1.15]
**Mediterranean**	46	0.802	0.747	[0.63, 0.87]	-2.533	[-3.71, -1.36]
**Germany (minus Trier and Aachen)**	38	0.766	0.743	[0.597, 0.889]	-2.334	[-3.67, -1.00]
**Central Continent**	30	0.826	0.846	[0.70, 1.00]	-3.391	[-4.70, -2.08]
**North Continent**	35	0.728	0.695	[0.57, 0.82]	-1.932	[-3.16. -0.70]

As seen in Table S1.3, the results for the socioeconomic urban regions are more varied, with scaling exponents exhibiting a wider range [0.695, 0.846]. While 7/10 fall squarely in the previously observed [0.72, 0.79] range, 3/10 fall outside of it. “North Continent” (n = 35), comprised of north German Hanseatic cities and the Low Countries, has a relatively low scaling coefficient of 0.695. Nevertheless, North Continent’s estimated scaling coefficient does not fall outside of the $(2/3 \leq \alpha \leq 5/6)$ interval, and is only 0.05 less than the median for all ten cases. This is an interesting result, as the combined region might be considered somewhat of a commercial maritime trading zone. Even more anomalous are the two strongly overlapping groupings “Northern and Central France” (n = 30) and “Central Continent” (n = 30) whose estimated scaling coefficients sit just *above* 5/6 (~0.01 over). These groupings also have very low estimated scaling prefactors. Given their lower level of spatial agglomeration, this suggests that these regions could be subject to hierarchical institutional dampening effects. The historical coherence of Central Continent might be questioned (straddling a long east-west band from Bavaria to Burgundy), and may therefore perhaps an artifact of system pooling and sampling error. Nevertheless, the historical coherence of the Northern and Central France grouping, and high R^2 (0.89), lends its result more credibility. Yet the fact that these two urban systems overlap in Alsace, Lorraine, and Burgundy/Franche-Comte/Dauphine (n = 14; roughly half of their datasets), and that other urban system delineations including these regions have higher scaling exponents (France and Belgium, and the Holy Roman Empire without Italy; see Tables S1.1 and S1.2, above), suggests that this sub-region may have a higher scaling exponent than others. Given the lack of sufficient sample size to analyze this sub-region by itself, this possibility must be left to future research.

S1.3 ANOVA of Scaling Parameters

At face value, the results of the analyses of alternative urban system delineations are fairly ambiguous. On the one hand, 3/10 socioeconomic regions expanded the range of scaling exponents beyond their original range, and 2/10 socioeconomic regions had estimated scaling coefficients just above the range predicted by the social reactor model. This might suggest that the range of scaling parameter variability evident in the data is meaningful. On the other hand, all

estimated scaling exponents were moderately-to-strongly sublinear (<0.85), all have widely overlapping 95% C.I.'s, 16/18 of the estimated scaling coefficients clustered tightly around ~0.73, and the remaining 2/18 urban systems strongly overlap geographically. This might suggest that the observed variability is insignificant, and probably due to sampling error (e.g. random error, system pooling, system partitioning, etc.).

To provide a more objective means of interpreting variability among the scaling parameters, we conducted an ANOVA (analysis of variance) of the scaling analysis results. In particular, the prefactor (intercept) and scaling coefficient (exponent) F-statistics of each *independent* set of urban regions (i.e. urban regions without any overlapping cases) were calculated and subjected to null hypothesis testing. Our assumed null hypothesis H_0 was that the true values of the scaling parameters for each urban system sample are identical (and therefore any variability in estimated parameter values is due to sampling error). Our alternative hypothesis H_1 was that the true values of the scaling parameters for each urban system sample are different. As such:

$$H_0: \sigma_1 = \sigma_2$$

$$H_1: \sigma_1 > \sigma_2$$

where σ_1 is the variance *between* urban system samples (SSB/df_1) and σ_2 is the variance *within* samples (SSW/df_2). We nominally use a one-tailed 5% rejection region ($\alpha = 0.05$) as a benchmark for evaluating each sample F-statistic. However, we had no intent to hold ourselves to this conservative level of risk of Type I error—since the "assumed" null hypothesis is the theory under scrutiny—and it should be noted that we carefully took into account the probability of obtaining each F-statistic (or greater) given its F-distribution and the assumption of the null hypothesis.

Table S1.4 presents the results of five of the estimated scaling exponent and prefactor F-tests for four independent sets of urban regions. The table does not include every single independent urban system set, but it does include the urban region sets most relevant to the hypothesis testing results.

In all cases the F values were very low, highlighting the similarity (low variability) among the estimated parameters, and the high variability within the samples. Because of the large within-group and small between-group degrees of freedom, the F-distributions are strongly skewed to the left. As seen in Table S1.4, the F-values necessary to exceed the one-tailed 5% rejection region range from about 2.5 to 3, yet the F-statistics exhibited by the data are all < 1.0. Correspondingly, assuming H_0 is true (that the true parameter values are identical), the probability of obtaining the observed F-statistics or higher due to random sampling error is extremely high, ranging from 0.57 to 0.99. Indeed, we would not be able to reject the null hypothesis even if we raised the decision level to $\alpha = 0.50$. Given this very high level of risk of Type I error, we must accept (fail to reject) H_0 for all estimated parameters and all sets of urban systems.

Thus, although some of the alternative urban system delineations appeared to suggest interpretable differences in estimated scaling parameters, our ANOVA clearly demonstrates that there is insufficient variability among them to venture to interpret their meaning. As stated above, it is probable that random error from the sample data. It is also possible that systematic

errors like system pooling and partitioning dominate the variability among certain datasets. Further exploration and resolution of these issues will have to wait until more data are available.

Table S1.4 Independent Urban System Set F-test results for estimated scaling exponents (red) and prefactors (blue)

Urban System Set	σ_1	σ_2	F	Critical F Value (α=0.05)	P	σ_1	σ_2	F	Critical F Value (α=0.05)	P
	Scaling Exponent					Scaling Prefactor				
**Original Set**<br>England<br>Fr. & Belg.<br>Germany<br>N. Italy	**0.049**	**0.135**	**0.361**	**2.658**	**0.78**	**0.673**	**11.959**	**0.056**	**2.658**	**0.98**
**Political A**<br>Capetian<br>Plantagenet<br>HRE (full)	**0.066**	**0.124**	**0.529**	**3.051**	**0.59**	**1.011**	**11.004**	**0.091**	**3.051**	**0.91**
**Political B**<br>Capetian<br>Plantagenet<br>HRE (no Italy)<br>N. Italy	**0.078**	**0.143**	**0.547**	**2.658**	**0.65**	**1.197**	**12.766**	**0.094**	**2.658**	**0.96**
**Socioecon A**<br>England<br>N. Fr. & Belg<br>S. France<br>Ger. (reduced)<br>N. Italy	**0.022**	**0.155**	**0.143**	**2.425**	**0.97**	**0.271**	**13.634**	**0.019**	**2.425**	**0.9992**
**Socioecon B**<br>Eng. (reduced)<br>N. Continent<br>N. & C. France<br>Mediterranean	**0.132**	**0.139**	**0.955**	**2.658**	**0.78**	**1.193**	**12.432**	**0.096**	**2.658**	**0.98**

Explanation of columns: σ_1 is the variance between urban system groups; σ_2 is the variance within urban system groups; F is σ_1/σ_2; P is the probability of getting the observed F-stat or greater by chance assuming H_0.

S1.4 Bibliography

1. D. Pumain, Settlement systems in the evolution. *Geografiska Annaler* **82 B**, 73 (2000).
2. A. Bretagnolle, D. Pumain, C. Rozenblat, Space-time Contraction and the Dynamics of Urban Systems. *Cybergeo: European Journal of Geography* **61**, (1997).
3. F. Braudel, *The Structure Of Everyday Life* Civilization And Capitalism, 15th-18th Century (Harper and Row, New York, 1979), vol. 1.
4. F. Braudel, *The Wheels of Commerce*. Civilization and Capitalism, 15th-18th Century (New York, Harper & Row, 1979), vol. 2.
5. A. Zeileis, Econometric Computing with HC and HAC Covariance Matrix Estimators. *Journal of Statistical Software* **11**, 1 (2004).
6. R_Core_Team. (R Foundation for Statistical Computing, Vienna, Austria, 2015).
