# Supplementary material for: Population-Area Relationship for Medieval European Cities
Source: PLoS One. 2016 Oct 5;11(10):e0162678. doi: 10.1371/journal.pone.0162678 (PMC5051806; doi:10.1371/journal.pone.0162678)
Supplement: S2 Appendix — This appendix includes tables of all historical population and settled area estimates that went into the database, the methods and reasoning behind each data point, a brief overview of how to read these tables, and a comprehensive bibliography of the sources. (PDF) [file pone.0162678.s002.pdf]

S2 Appendix: Database Construction

S2.1 Overview......1
S2.2 England Population Estimates......1
S2.3 Northern Italy Population Estimates......2
S2.4 Population Estimate Tables......3
S2.5 Settled Area Estimate Tables......9
S2.6 Bibliography......20

S2.1 Overview

The tables in this appendix include the side-by-side quantitative figures used to estimate the population and settled area of each of the 173 settlements analyzed in this paper (numbered 1-173). Tables 1-4 (in S2.4, below) are population estimates organized by urban region, and tables 5-8 (in S2.5, below) are settled area estimates organized by urban region. Each row in the tables represents a settlement, and the columns organize the various sources. The citation and date (or approximate time period) corresponding to each quantitative figure is indicated in either the column heading of the table or in its particular cell. The two leftmost columns of each table include the final population or settled area estimate for each settlement, as well as the method and reasoning behind the selection of the final estimate. Because many cases require a longer explanation than can be included in the table, the methods, reasoning, and any further citations used in their estimation have been included as numbered lists below each table. A bibliography of all sources used follows these tables.

However, as noted in the "Methods and Materials" section of the main text, the population estimates for English and Italian cities *ca.*1300 were taken directly from Campbell (2008) and Malanima (2005), respectively. Although we include alternative population figures for Italian and English cities in their respective tables below for the sake of comparison, only the population estimates of Campbell (2008) and Malanima (2002; 2005) were used. As such, the population tables for England and Italy do not include "Final Estimate" or "Reasoning" columns. Instead, the reasons for our use of these sources are elaborated upon in sections S2.2 and S2.3, followed by the tables in S2.4 and S2.5.

S2.2 England Population Estimates

The urban historical demography of fourteenth-century England is unique because of the existence of nation-wide tax records. Whereas the cities of other European regions primarily have asynchronous and idiosyncratic records for individual cities, the nation-wide English taxes sought to assess the same phenomena for all cities at the same time. The most accurate of these tax records is the 1377 poll tax, which has commonly been extrapolated to make estimates

c.1377 (at least since Russell's seminal 1948, *British Medieval Population*)—by multiplying the number of taxpayers by a historically informed constant, thereby correcting for the proportion of the population not assessed by the tax (see A. Dyer, 1995; Fenwick, 1998; Goldberg, 1990; Hatcher, 1977; Hettinger, 2000; Kermode, 1999; Kowaleski, 1995; Rigby, 2010). In contrast, heterogeneous pre-plague urban population estimates for different English cities variously make use of the 1377 poll tax rolls, retrodictions thereof (based on estimations of how much a city's population declined due to plague c.1348-1377), the early fourteenth century lay subsidies (also tax records), and local idiosyncratic historical records from particular towns (see, e.g. Keene & Rumble, 1985; C. Dyer and Slater 2000; Kermode, 2000; Nightingale, 1996; Kowaleski, 1995; Rutledge, 1988; 1995; 2004). However, some of the most recent, well researched, and most methodologically systematic population estimates for pre-plague England have been produced for c.1290 by Campbell (2008). Campbell's (2008) urban population estimates not only make use of systematic 1377 poll tax retrodictions, but also extrapolation estimates from the number of taxpayers in the newly researched 1327/1332 lay subsidies (also tax rolls). Campbell averages the 1377 retrodictions and 1327/1332 estimates into a single figure for each town, and then corrects inaccurate figures produced by this method by replacing them with more reliable population estimates made by historical experts of particular towns. Because Campbell's (2008) method is both consistent with other leading estimates, and it incorporates new evidence in a systematic way, we relied on Campbell's dataset rather than producing our own.

S2.3 Northern Italy Population Estimates

Unlike England, the population estimates of Medieval Northern Italy are more heterogeneous in source as well as the modern literature—both of which are widely varied, and contain a great range of population estimates. Our compilation and evaluation of Italian population estimates preceded our discovery of Malanima's (2005) city population database, following the method outlined in the "Methods and Materials" section of the main text. When we compared our dataset to Malanima's (2005) most of the population estimates were the same or similar because we had both (A) used Bairoch et al.'s (1988) data as a principle baseline source, and (B) used contextual historiographical criticism to evaluate the range of estimates. However, certain other Because Malanima's (2005) database used a much wider range of sources (Malanima, pers. comm.), and because the author is an expert in the field, we decided to use the Malanima (2005) database for our Northern Italy population figures.

S2.4 Population Estimate Tables

England City Population Estimates (in thousands)

ID#	Name	Bairoch et al. c.1300 (1988: 32-35)	Russell Pre-Plague (1972: 124)	Other Sources	Campbell c.1290 (2008: 908-9)
1	London & Southwark	35	60	80-100 (PP) (Dobson, 2000: 275);<br>60 (PP) (Nightingale, 1996: 97-8)	70
2	York	8	18	>12 (PP) (Dobson, 2000: 275)	22.7
3	Bristol	11	16	>12 (PP) (Dobson, 2000: 275)	14.4
4	Lincoln	9	8.9		12.3
5	Norwich	13	13	25 (PP) (Rutledge, 1988; 1995; 2004)<br>4-5 (PP) (Brodt, 2000: 654)	14
6	Newcastle-upon-Tyne	8			9.9
7	Oxford				9.8
8	Coventry	12	12		9.5
9	Canterbury				8.8
10	Salisbury	8	8.1		7.7
11	Gloucester				7.2
12	Great Yarmouth				70
13	Cambridge				6.9
14	King’s Lynn	8	7.8	8-10 (PP) (Brodt, 2000: 654)	6.9
15	Winchester	15		10-12 (PP) (Miller & Hatcher, 1995: 263)	9.5
16	Scarborough				6.5
17	Colchester	7	7.4		3.5
18	Boston	7	7.2		6.2
19	Bury St Edmunds				5.8
20	Shrewsbury				5.7
21	Hereford				5.5
22	Leicester	3			5.4
23	Ipswich				5.3
24	Stamford			5 (PP) (Dyer & Slater, 2000: 632)	4.5
25	Northampton				4.2
26	Nottingham	4			4.2
27	Plymouth	12	12		3.8
28	Kingston-upon-Hull				3.8
29	Exeter				3.7
30	Worcester				3.7
31	Southampton				3.5

32	Ely				3.5
33	Chester				3.3
34	Ludlow				3.3
35	Lichfield				2.7
36	Newark				2.7
37	Durham				2.6
38	Bridgnorth				2.4
39	Pontefract				2.4
40	Doncaster				2.3

Greater France and Belgium City Population Estimates (in thousands)

Years are either in the column heading or in the estimate boxes; PP= pre-plague

ID#	Name	Bairoch et al. c.1300 (1988: 11-2, 23-31, 67)	Russell Pre-Plague (1972: 148-62)	Chandler & Modelski (1987: 143-75)	Carpentier & Le Mene (1996: 313-5)	Nicholas (1997: 178-81)	Other Sources	Final Estimate	Reasoning
41	Paris	150	80	274 (1328)	210 (1328)	200 (1300); 275 (1328)	210 (1330)Grantham (2012: 62) 200 (1300) Geremek (1987: 67)	200	(see below)
42	Rouen	35	34	50 (1300)	>40 (1300)	40 (1300)		40	(see below)
43	Orleans	10	22.5	36 (1300)	10-20 (PP)	25 (1300)		21.7	(see below)
44	Reims	14	19	15 (1325)	20 (PP)	20 (1300); 15 (1328)		20	(see below)
45	Beauvais	16	15.5			10-20 (1300)		15.5	(see below)
46	Troyes	25	14.8	20 (1300)	25			25	Mode
47	Chalons-sur-Marne	10	10					10	Mode
48	Chartres	7	9		10-20 (PP)			8.7	(see below)
49	Sens	5	5					5	Mode
50	Provins	10						10	Only Est.
51	Nancy	1						1	Only Est.
52	Ghent		56	42 (1309)			60 (1300) Van Bavel (2010: 281) 64 (1300) Stabel (1997: 31)	60	(see below)
53	Bruges	40	30	50 (1300), 35 (1340)	35 (PP)	35 (1340)	45 (1300) Stabel (1997: 31)	45	(see below)
54	Tournai	20	20	20 (1385)	10-20 (PP)	20 (1300)	50 (PP) Stabel (1997: 69)	35	(see below)
55	Ypres	30	14	30 (1311)	20 (PP)		30 (1300) Van Bavel (2010: 281) 30 (1300) Stabel (1997: 33)	30	Mode

56	Saint-Omer	35			35 (PP)		30 (1300) VanBavel (2010: 281)<br>>30 (1300) Stabel (1997: 65)	35	Mode
57	Mons	6	10				10 (1300) Stabel (1997: 69)	10	Mode
58	Calais						14 (1300) Rose (2008: 8)<br>10-12 (1300) Nicholas (1992: 196)	12	(see below)
59	Amiens	21	21			20 (1300)		21	Mode
60	Arras	30		20 (1300)	20 (PP)	10-20 (1300)	30 (1300) Van Bavel (2010: 281)<br>30 (1300) Stabel (1997: 65)	30	Mode
61	Lille	30		24 (1382)	10-20 (PP)	20 (1300)	30 (1300) Van Bavel (2010: 281)<br>30 (1300) Stabel (1997: 65)	30	Mode
62	Liege	11	11	25 (1300)				15	Mean
63	Namur		14.4					14.4	Only Est.
64	Mechlin	10	10					10	Mode
65	Tours	25	26.3		10-20 (PP)	30 (1300)		24	(see below)
66	Blois	5	5					5	Mode
67	Bourges	16	16.3		10-20 (PP)	10-20 (1300)		16.3	(see below)
68	Poitiers	15	15		10-20 (PP)			15	(see below)
69	Toulouse	30	35	35 (1335)	30 (1335)	35 (1300)	30 (1335) Reyerson (1998: 253)	33	Mean
70	Bordeaux	30	20	30 (1300)	30 (1335)	20 (1300)		30	Mode
71	Albi	7	10.7		10-20 (PP)			10.7	(see below)
72	Perigueux	7	6		8-9 (PP)			8	(see below)
73	Agen	6	6					6	Mode
74	Angouleme	5	5					5	Mode
75	Rodez	5	5					5	Mode
76	Limoges	4	4					4	Mode
77	Cahors	5	4					4.5	Mean
78	Tarbes	4	4					4	Mode
79	Pamiers	4	3.5					3.5	(see below)
80	Montpellier	35	40	35 (1300)	35-40 (PP)	40 (1300)	40 (1340s) Caille (1998: 60)<br>35-40 (1300) Reyerson (1998:254)	40	Mode
81	Narbonne	30	25	30 (1300)	30 (1335)	25 (1300)	30000 (1340s) Caille (1998: 60)	30	Mode
82	Avignon	30	18	35 (1348)	30-40<br>(1340s)	10-20 (1300)	6000 (1300)<br>Rollo-Koster (1998: 73-4)	23.2	(see below)
83	Beziers	16	14.5	14.5 (1304)	10-20 (PP)			15	(see below)
84	Marseilles	31	12	20 (1300)	10-20 (PP)			19.5	(see below)
85	Arles	8	8.4		15 (PP)		5 (C14) Reyerson (1998: 253)	6.7	(see below)
86	Aix-en-Provence	6	6		15 (PP)		6000 (1330s)<br>Reyerson (1998: 253)	6	Mode
87	Sisteron	6	5.6					5.6	(see below)
88	Lodeve	4						4	Only Est.
89	Toulon	2.8		3 (1314)			3500 (PP) Barnell (1998:239)	3.1	Mean

90	Carcassone	11	9.5		10-20 (PP)			10	(see below)
91	Dijon	17	17	18 (1320)				17.3	Mean
92	Lyon	20	10.5	35 (1300)	20 (1320)	10-20 (1300)		20	Mode
93	Besancon	8	8		9-10.5 (PP)	10-20 (1300)		10.2	(see below)
94	Autun	7	7					7	Mode
95	Le Puy	6	6					6	Mode
96	Valence	5	5					5	Mode
97	Clermont-Ferrand	5	5		10-20 (PP)			8.3	(see below)
98	Vienne	4	5					4.5	Mean
99	Macon	4	4					4	Mode
100	Geneva	4	3					3.5	(see below)
101	Chalon-sur-Saone	3	3					3	Mode
102	Grenoble	3	3					3	Mode
103	Metz	30		32 (1300)	25 (PP)	20 (1300)		26.8	Mean

41. Paris: More recent historical demography has progressively revised the population of the Capetian capital upwards into the 200,000 range (see, e.g., Cazelles, 1972; Carpentier & Le Mene, 1996) based on revised (literal) philological interpretation of ‘hearths’ as ‘households,’ rather than as ‘individuals’ (early 20th Century historians could not believe the size of the historically reported figures). For this reason, Russell (1972) is discounted. The mean of the remaining figures is 197,700, which we rounded to an even 200,000. Roughly in the middle of the range of estimates, we find this approximation to be reasonable.
42. Rouen: The mean of all five estimates is 39,800, which we rounded to an even 40,000 given the two estimates at that number.
43. Orleans: Mean of all estimates, using the midpoint of the Carpentier & Le Mene (1996) range (15,000)
44. Reims: The population of Reims experienced population decline from epidemic disease c.1320 that was not recovered from before the Black Plague (Nicholas, 1997: 276). After removing the 14-15,000 estimates from to the later period, we used the mode of 20,000 as the final estimate.
45. Beauvais: Mean of all estimates, using the midpoint of the Nicholas (1997) range (15,000)
48. Chartres: Using the minimum of the Carpentier & Le Mene (1996) range, the mean of the three estimates was 8,666, which we rounded to an even 8,700.
52. Ghent: More recent estimates have revised Ghent’s population upwards from the 40,000 range, so Chandler and Modelski (1987) is discounted. The Averge and Median of the remaining estimates is 60,000.
53. Bruges: Given that the 30-35,000 population estimates are for the mid-14th century, the average and median of the remaining estimates is 45,000.
54. Tournai: Despite the general consensus of the sources at around 20,000, Stabel (1997: 69, n.25) cites the more recent and authoritative work of Dury (1986) which puts the population of Tournai at 50,000 on the eve of the Plague. As such, we have used the midpoint of 50,000 and 20,000 to serve as the population estimate c.1300.
58. Calais: Rose (2008) suggests that the 14,000 estimate from Derville and Vion (1985) is a bit on the high side, so we used the midpoint of 12,000 within Nicholas’ (1992) range of 10-12,000.
65. Tours: Mean of all estimates, using the midpoint of the Carpentier & Le Mene (1996) range (15,000)

67. Bourges: Russell (1972) and Bairoch et al. (1988) both cite the same source, which was rounded down by Bairoch et al. (1988). Given that 16,300 is within the two other range estimates, it was used as the final estimate.
68. Poitiers: The mode of 15,000 is within the range estimated by Carpentier & Le Mene (1996)
71. Albi: The estimate by Russell (1972) better fits the range specified by Carpentier & Le Mene (1996), and thus was selected.
72. Perigueux: minimum of the Carpentier & Le Mene (1996) range selected, as it is only 1,000 away from Bairoch et al. (1988)
79. Pamiers: Russell (1972) and Bairoch et al. (1988) both cite the same source, which was rounded up by Bairoch et al. (1988)
82. Avignon: Mean using midpoints of estimated ranges, rounded to nearest hundred
83. Beziers: Mean of all estimates, using the midpoint of the Carpentier & Le Mene (1996) range (15,000)
84. Marseilles: Mean of all estimates, using the midpoint of the Carpentier & Le Mene (1996) range (15,000)
85. Arles: Given the discussion in Reyerson (1998), and the authority of her recent sources, 15,000 is much too high for Arles c.1300. The Bairoch et al. (1988) estimate is simply the rounded Russell (1972) estimate, so we averaged Russell (1972) and Reyerson (1998).
87. Sisteron: Russell (1972) and Bairoch et al. (1988) both cite the same source, which was rounded up by Bairoch et al. (1988)
90. Carcassone: Bairoch et al. (1988) estimate fits in Carpentier & Le Mene (1996) range
93. Besancon: Mean of all estimates, using the midpoint of ranges
97. Clermont-Ferrand: Mean of all estimates, using the midpoint of the Carpentier & Le Mene (1996) range (15,000)
100. Geneva: Russell (1972) and Bairoch et al. (1988) both cite the same source, but reported them differently, so the mean was taken

Northern Italy City Population Estimates (in thousands)

ID#	Name	Bairoch et al. c.1300 (1988: 40-9)	Russell (1972: 44, 64, 68)	Chandler & Modelski (1987: 107-24)	Balchin (2008:188)	Nicholas (1997:178-81)	Other Sources	Malanima c.1300 (2005: 1-7)
104	Bergamo	14	14 (PP)					12
105	Brescia	24	48 (PP)	24 (1300)				45
106	Como	12	12.3 (PP)					12
107	Cremona	40	44 (PP)	38 (1300)				45
108	Mantova	30	30 (PP)			34 (1300)		30
109	Milano	100	75 (PP)	60 (1300)	150 (1300)	100 (1300)	150-200 (Wickham, 2015: 112)	150
110	Monza	10	9.6 (PP)					9
111	Pavia	30	30 (PP)	30 (1300)		35 (1320)		20
112	Padova	35	33 (PP)		38 (1320)			40
113	Venezia	110	100 (PP)		120 (1338)	100 (1300)		110
114	Verona	30	40 (PP)		38 (1325)	34 (1300)		40
115	Vicenza	22	22 (PP)					20
116	Genova	100	60 (PP)	100 (1300)	60 (1290)	100 (1300)		60
117	Bologna	40	60-70 (PP)		54 (1320)			50
118	Faenza	12	11.6 (PP)					10
119	Ferrara	36	17 (PP)					12

120	Forli	14	13.8 (PP)					14
121	Modena	18	18 (PP)					19
122	Parma	22	22 (PP)					25
123	Piacenza	20	20 (PP)	24 (1316)		30 (1300)		23
124	Ravenna	12	11.5 (PP)					12
125	Reggio Emilia	14	13.5 (PP)					13
126	Rimini	13	13.4 (PP)					14
127	Arezzo	20	20 (late C13)					18
128	Firenze	95	96 (late C13)	60 (1300)	95 (1338)	100 (1300)		110
129	Pisa	38	38 (late C13)		38 (1293)	38 (1293)		30
130	Pistoia	11	11 (late C13)					12
131	Prato	15	9 (late C13)					13
132	Siena	50	52 (late C13)		52 (1328)			50
133	Lucca	16	23 (late C13)	18 (1300),<br>23 (1333)				25

Germany City Population Estimates (in thousands)

ID#	Name	Bairoch et al. c.1300 (1988:4-9, 67)	Russell Pre-Plague (1972:80-108)	Chandler & Modelski (1987: 195-209)	Other Sources	Final Estimate	Reasoning
134	Augsburg	25	25			25	Mode
135	Bamberg	8	8			8	Mode
	Bautzen	3	8			5	Average
136	Bremen	12	12			12	Mode
137	Dresden	5	5			5	Mode
138	Erfurt	30	10	30 (1300)		23.3	Average
139	Frankfurt am Main	13	12	13 (1300)		13	Mode
140	Goerlitz	5	9			7	Average
141	Greifswald	8	8			8	Mode
142	Halberstadt	6	6			6	Mode
143	Hamburg	8	9	8 (1311)	5 (1300) (Nicholas, 2003: 19)	8	Mode
144	Hannover	5	5			5	Mode
145	Leipzig	3				3	Only Estimate
146	Luebeck	28	28	22 (1300)	25 (1300)(Nicholas, 2003: 19)<br>15 (1300)(Leguay, 2000: 104-5)	23.6	Average
147	Lueneburg	8	8			8	Mode
148	Mainz	25	10	24 (1300)		24.5	(see below)
149	Noerdlingen	10	10			10	Mode

150	Nuernberg	12	14	13 (1363)	20 (1300) (Ammann, 1967: 409)	13	Average
151	Paderborn	7	7			7	Mode
152	Regensburg	11	11			11	Mode
153	Rostock	14	14			14	Mode
154	Stralsund	12	12			12	Mode
155	Stuttgart	5				5	Only Estimate
156	Ulm	4	8	10 (1377)		7.3	Average
157	Wismar	8	8			8	Mode
158	Worms	20	16	20 (1300)		18.7	Average
159	Wuerzburg	7	7			7	Mode
160	Braunschweig	12	12			12	Mode
161	Muehlhausen		7.5			7.5	Only Estimate
162	Strasbourg	15	25	15 (1300)	20 (1300) (Ammann, 1967: 409)<br>25 (Nicholas, 1997: 178-81)<br>10-20 (Carpentier & Le Mene, 1996: 313-5)	20	(see below)
163	Basel		11			11	Only Estimate
164	Konstanz	6				6	Only Estimate
165	Zurich	6	13			9.5	Average
166	Aachen	21	18	21 (1300)		20	Average
167	Dortmund	7	7			7	Mode
168	Koeln	54	40	54 (1300) 57 (1333)	80 (Nicholas, 2003: 19)	54	Mode
169	Muenster	16	16			16	Mode
170	Osnabrueck	9	9			9	Mode
171	Soest	12	8	14 (1300)		11.5	Average
172	Trier	11	10.5	15 (1300)		12.7	Average

148. Mainz: Average of more recent estimates, excluding earlier Russell (1972) outlier

162. Strasbourg: Mean and Median of the 5 single point estimates, which fit with the estimated range by Carpentier and Le Mene (1996)

S2.5 Population Estimate Tables

England City Settled Area Estimates (in hectares)

ID #	Name	Kermode (2000: 442-3)	Barley (1976: 61-7)	Russell (1972: 124)	Russell (1958: 61)	Keene (1976:72-80)	Other Sources	Final Estimate	Reasoning
1	London & Southwark			288	288 (C14)	330		330	(see below)
2	York	106.4		94	84 (late C13)	145	135 (early C14) Palliser (2014: 130-1)	135	(see below)
3	Bristol	55		80+		130		130	(see below)
4	Lincoln	54.5		67	67 LM	115		115	(see below)
5	Norwich	388.6	219	85	85 C13		185 (early C14) Rawcliffe (1999: 3)	185	(see below)
6	Newcastle-upon-Tyne	64.7	65		90 C14			90	(see below)
7	Oxford	38	40				94 (early C14) Steane (2014: 120)	94	(see below)
8	Coventry	85	71					85	(see below)
9	Canterbury	48.5	62		40 C14	125 late C12		110	(see below)
10	Salisbury	83	80	72				83	(see below)
11	Gloucester	52	55			75		75	Keene map includes suburbs
12	Great Yarmouth	54					80 (C14) Aston & Bond (1976: 95)	80	(see below)
13	Cambridge	63					88 Rubin (1987: xiv)	88	(see below)
14	King’s Lynn	121	121	70				95	(see below)
15	Winchester	58	60		55 LM	131 mid-C12		100	(see below)
16	Scarborough		60					60	Only Estimate
17	Colchester	44	45	60+				60	(see below)
18	Boston	20	20				walled area doubles with suburbs Butler (1976: 42)	40	(see below)
19	Bury St Edmunds						78 (1295) Gottfried (1982: 27, 35)	78	Only estimate
20	Shrewsbury						58 (C14) Carver (1987: 61)	58	Only Estimate
21	Hereford	37.6				75		75	(see below)
22	Leicester	40.5			40-50 LM	75		75	(see below)

23	Ipswich						90 (1300) Amor (2011: 14-15)	90	Only Estimate
24	Stamford		64					64	Only Estimate
25	Northampton					90		90	Only Estimate
26	Nottingham						63 Beresford & St. Joseph (1979:177)	63	Only Estimate
27	Plymouth			72				72	Only Estimate
28	Kingston-upon-Hull				33 C14		35 Butler (1976: 42)	35	(see below)
29	Exeter	40	40					40	Only Estimate
30	Worcester						40 (C14) Baker & Slater (1992: 50)	40	Only Estimate
31	Southampton		20				35 (C14) Platt (1976: 153)	35	(see below)
32	Ely						57 (C14) Atkinson et al. (2002: 28-33)	57	Only Estimate
33	Chester		53					53	Only Estimate
34	Ludlow		20				41 (C14) Hindle (1990: 56)	41	(see below)
35	Lichfield						30 (C14) Schofield & Vince (2003: 168)	30	Only Estimate
36	Newark		50					50	Only Estimate
37	Durham		25					25	Only Estimate
38	Bridgnorth						20 (C12 walls) Halsam (n.d.: 2-11)	20	Only Estimate
39	Pontefract						40 (C14) Aston & Bond (1976: 80)	40	Only Estimate
40	Doncaster						25 (C14) Buckland, Magilton & Hayfield (1989: 48, 59)	25	Only Estimate

1. London: According to King (1983v1: 272), the walls of London were completed by c.1312 and were never expanded afterwards because the city's suburbs continued to grow uncontrollably. The walled area itself was only about 200 ha (Keene, 1976; Barron, 2000; Barley, 1976). Russell (1958; 1972) implies that his 288 ha area estimate for London applies to the 14th century walled area, which is based on the analogy with a 1572 map of London—a period with a very similar population (about 80,000) and spatial distribution (De Vries, 1984; Palliser, 1992; Barron, 2000; Keene, 1976). Keene (1976) echoes this analogy, basing his own suburban map of London off of the 1572 map. Measurement of Keene's (1967) map suggests a total settled area of about 330 ha.
2. York: Palliser (2015) map most recent and reliable estimate
3. Bristol: Keene's (1976) map includes suburban sprawl, and measures a 130 ha settled area and 55 ha walled area—perfectly tying together Kermode's (2000) 55 ha walled area estimate and Russell's (1972) 80+ ha settled area estimate
4. Lincoln: According to King (1983v1: 226 n.50), Lincoln had suburbs c.1300. Kermode's (2000) walled area estimate 54.4 ha excludes the suburbs, while Russell's (1972) 67 ha is attributed to the "late medieval" period, and thus likely after the plague. Keene's (1975: 105) map of the C12 walled town and built-up suburbs of Lincoln indicate a roughly 55 ha walled area, and 130 ha including the suburbs. Keene (1976) also notes that the suburbs contracted from their peak C13 extent during the C14, suggesting that the map's settled area is too large for c.1300. For this reason we reduced the total settled area to 115 ha.
5. Norwich: Norwich did not have city walls before their construction c.1294-1342 (King, 1983v2: 308, 311). Russell (1972, 1958) estimates Norwich's area to be 85 ha, which he applies liberally to both the 13th century (1958) and the pre-plague 14th century (1972). Russell's (1958) source is Stephenson (1937), whose map explicitly dates to the 13th century, and should not be applied to the expanded 14th century because of its massively expanding population (see, e.g., Britnell, 2000; Kermode, 2000; Rutledge, 1988; 1995; 2004). Kermode's (2000) measurement of the area enclosed by

Norwich's walls is 388 ha, but this 388 ha area is far greater than 219 ha maps of the wall-enclosed area produced by Barley (1976) and Rawcliffe (1999). The walls enclosed a considerable amount of marshland, so the 14th century settled area was considerably than the wall-enclosed area (Schofield and Stell, 2000). Using a map of Norwich produced by the Norfolk Archaeological Unit, the non-marsh intra-mural area measures about 185 ha (Rawcliffe, 1999).

6. Newcastle: Newcastle had suburbs c.1300 (King, 1983v2: 311), and both Barley (1976) and Kermode (2000) are walled areas. As such, we used Russell's (1972) 90 ha settled area for Newcastle in order to take into account the f extra-mural suburban sprawl
7. Oxford: Kermode's (2000) walled area estimate for Oxford is 38 ha, and the map provided by Barley (1976) indicates a walled area of 40 ha. According to King (1983v2: 388), this wall circuit was completed in the mid-13th century, and Keene (1976) notes the importance of Oxford's suburban areas. As such, we used Steane's (2014) 94 ha map of Oxford's intra- and extra-mural settled area.
8. Coventry: The 85 ha walled area reported by Kermode (2000) was constructed in the 1350's and enclosed the town's pre-plague suburban sprawl (Schofield and Stell, 2000). It is thus unclear what period the 71 ha Barley (1976) map refers to, and suggests that the Kermode 85 ha estimate applies to a pre-plague settled area.
9. Canterbury: The 40 and 48.5 ha estimates are wall-enclosed areas, and Keene's (1976) map of Canterbury's total settled area c.1200 measures 125 ha including its extramural suburbs. However, Keene (1976) also notes that the suburbs contracted from their peak C13 extent during the C14, suggesting that the map's settled area is too large for c.1300. For this reason we reduced the total settled area to 110 ha.
10. Salisbury: Russell (1972) and Kermode (2000) provide slightly divergent area estimates for Salisbury—72 ha and "c.83" ha, respectively—and Barley (1976) provides a walled area map that measures roughly 80 ha. Given that Barley's walled area is closer to Kermode's, and the town probably had some extra-mural suburban sprawl, we chose Kermode's (2000) larger 83 ha estimate for the settled area c.1300.
12. Yarmouth: Kermode (2000) suggests that the 1320s walled area of Yarmouth was "c.54 ha," but the pre-plague walled area map provided by Aston and Bond (1976) measures 80 ha. Since Yarmouth had a major fishing suburb (Gorleston), the larger 80 ha seems a better settled area estimate for Yarmouth.
13. Cambridge: The circular earthen defense perimeter around Cambridge was defined in 1267 (King, 1983v1: 41), and Kermode (2000) estimates that the defensive perimeter around Cambridge enclosed roughly 63 ha. Nevertheless, Keene (1976) notes that Cambridge had a single populous extra-mural suburb in the thirteenth century, extending towards Barnwell to the east. Using Rubin's (1987) map of Cambridge c.1445 to measure the eastern suburb, the central town nucleus itself, and the castle zone to the north encloses 88 ha, which we use the settled area to reflect the town's more extensive C14 layout.
14. Lynn: Russell's (1972) 70 ha settled area estimate for Lynn lies in sharp contrast to both Kermode's (2000) walled area estimate of "c.121" ha and Barley's (1976) walled area of 121 ha. As such, I have averaged 121 ha and 70 ha to come to a provisional settled area estimate of roughly 95 ha for Lynn c.1300.
15. Winchester: The estimates of Russell (1958, Barley (1967), and Kermode (2000) all correspond to the town's wall-enclosed area. According to Keene's (1975) map of medieval Winchester, its total settled area including extramural suburbs comprised 131 ha during the mid-12th century. However, Keene (1975) also notes that town suburban sprawl was in decline by about 1300, suggesting that Winchester's settled area was less than 131 ha. Since the town's prosperity and suburban sprawl peaked in the early to mid C12 (Keene, 1975; Campbell, 2008), we modified Keene's map downwards to 100 ha to better reflect Winchester's total settled area c.1300.
17. Colchester: Kermode (2000) estimates exactly 44 ha including the suburbs, and Russell (1972) estimates 60+ ha. However, Barley's (1976) map of Colchester's walled area indicates roughly 45 ha, suggesting that Kermode's information is either bad or a typo (i.e. mistakenly printing "including"

instead of “excluding”). Regardless, Russell’s (1972) base figure of 60 ha seems like a reasonable estimate, given that Kermode (2000) indicates the existence of a suburb.

18. Boston: Kermode (2000) estimates that the wall enclosed area of Boson was “c.20 ha,” and the walled area provided by Barley (1976) measures 20 ha, bounded by walls and a river. Although it has no scale, Butler’s (1976) map indicates that suburbs roughly doubled Boston’s settled area across the river, so we estimate 40 ha as the town’s settled area.
21. Hereford: Keene (1975) map includes suburbs
22. Leicester: Keene (1975) map includes suburbs
23. Kingston: Both estimates are very close, defer to we defer to our measurement from Butler (1976)
31. Southampton: Map in Platt (1976) includes suburbs
34. Ludlow: Hindle (1990) map includes suburbs

Greater France and Belgium City Settled Area Estimates (in hectares)

ID#	Name	Russell (1972: 148-62)	Russell Pre-Plague (1958: 61)	Nicholas (1997: 184-5)	Chandler & Modelski (1987: 143-75)	Other Sources	Final Estimate	Reasoning
41	Paris	237-437	378 (1292)		439 (1367)	800 w/suburbs Pounds (2005: 27);<br>817 w/suburbs Geremek (1987:67,88)	800	(see below)
42	Rouen	224					224	Only Estimate
43	Orleans	150					150	Only Estimate
44	Reims	196	196 (1358)		250 (1200s)	320 with suburbs (1200)<br>Heers (1990: 196)	240	Mean
45	Beauvais	103					103	Only Estimate
46	Troyes	99			102 (1125)	had suburbs c.1300<br>(Nicholas, 1997b: 72-6; 2003: 70)	120	(see below)
47	Chalons-sur-Marne	100					100	Only Estimate
48	Chartres	54-60				58 (1182) Heers (1990: 193)	60	(see below)
49	Sens	32					32	Only Estimate
50	Provins					146 (C14) (Garrigou-Grandchamp & Mesqui, 1991)	146	Only Estimate
51	Nancy					11 including suburbs (C14)<br>(Fray, 1997)	11	Only Estimate
52	Ghent	644	644 (late C14)		644 (1300),<br>80 (1100)		362	(see below)
53	Bruges	430			70 (1089)	400 (C14) Nicholas (1997b: 79)	300	(see below)
54	Tournai	175					175	Only Estimate
55	Ypres	112	112		112	385 walled area; 250 settled area<br>Jehel & Racinet (1996: 414)	250	(see below)
56	Saint-Omer					120 (Rose, 2008: 8)	120	(see below)

57	Mons	150					150	Only Estimate
58	Calais					50 (Rose, 2008: 8)	50	Only Estimate
59	Amiens	140				100 walled area only Pounds (2005: 148)	140	(see below)
60	Arras					182 with suburbs (C14) Jehel & Racinet (1996: 72)	182	Only Estimate
61	Lille			80 walled area (late C13)		120 Nicholas (1997b:76; 2003:70)	120	(see below)
62	Liege	248	80			200 (1300) Stiennon (1991: 12-4)	200	(see below)
63	Namur	75					75	Only Estimate
64	Mechlin	105					105	Only Estimate
65	Tours	175			130+ 2 suburbs (1354)		175	(see below)
66	Blois	32					32	Only Estimate
67	Bourges	115	115 (1180-1223)				115	Only Estimate
68	Poitiers	200			200 (1100)		200	Mode
69	Toulouse	289	212 (C13-C14)				250.5	Mean
70	Bordeaux	120	275 (1297-1326)				197.5	Mean
71	Albi	100	100			98 including suburbs (C14) (Biget, 1983)	98	(see below)
72	Perigueux	17.5+	40			70 including suburbs (C14) (Higounet-Nadal, 1984)	70	(see below)
73	Agen	57				60 including suburbs (C14) (Clemens, 1985)	60	(see below)
74	Angouleme	40					40	Only Estimate
75	Rodez	21+	21 w/o sububs (1350)			35 including suburbs (C14) (Suau, 1983)	35	(see below)
76	Limoges	32-50				75 including suburbs (C14) (Barriere, 1984)	75	(see below)
77	Cahors	25				45 including suburbs (C14) (Lartigaut, 1983)	45	(see below)
78	Tarbes	32-48				38 including suburbs (C14) (Berth et al., 1982)	38	(see below)
79	Pamiers	33					33	Only Estimate
80	Montpellier	100		45 walls only		150 including suburbs (C14) Caille (1998: 63)	150	(see below)

81	Narbonne	70+		37 walls only		150 including suburbs (C14) Caille (1998: 64)	150	(see below)
82	Avignon	151	151 (1351)		45 walls only (1200)		151	(see below)
83	Beziers	99	45 (C12)				99	(see below)
84	Marseilles	84					84	Only Estimate
85	Arles	36				40 (C12) Jehel & Racinet (1996: 312)	40	(see below)
86	Aix-en-Provence	40	42				42	(see below)
87	Sisteron	60					60	Only Estimate
88	Lodeve		55 (C14)				55	Only Estimate
89	Toulon	18					18	Only Estimate
90	Carcassone	68	40 (1359)				68	(see below)
91	Dijon	104					104	Only Estimate
92	Lyon	72					72	Only Estimate
93	Besancon	99					99	Only Estimate
94	Autun	80					80	Only Estimate
95	Le Puy	50					50	Only Estimate
96	Valence	40					40	Only Estimate
97	Clermont-Ferrand	38					38	Only Estimate
98	Vienne	36	36				36	Only Estimate
99	Macon	36					36	Only Estimate
100	Geneva	45					45	Only Estimate
101	Chalon-sur-Saone	24					24	Only Estimate
102	Grenoble	20	20 (C14)				20	Only Estimate
103	Metz			160 (1226)	159		160	(see below)

41. Paris: The figures from Russell (1958; 1972) and Chandler & Modelski (1987) intend to include Paris' extensive suburban sprawl by estimating the c.1300 settled area as the walled area of later periods (after the former suburbs had been enclosed. However, according to Geremek (1987: 67), the 439 ha wall built in the C15 only enclosed one-third of the C14 suburban sprawl's extent. If the C13 wall enclosed 250 ha, than the total c.1300 settled area of Paris was about 817 ha. Cross-referencing the suburb location map from Geremek (1987: 88) and the city walls map from Pounds (2005: 27), we measured an estimated total settled area of 800 ha. Given the similarity of these two figures, we have chosen 800 ha as our final estimate.
46. Troyes: The figures from Russell (1972) and Chandler & Modelski (1987) correspond to the town's walled area, but Troyes had extramural suburbs c.1300 (Nicholas, 1997b: 72-6; 2003: 70). As such, we have raised the town's settled area figure to 120 ha to compensate for these suburbs.
48. Chartres: The estimate range by Russell (1972) corresponds to the C12 wall-enclosed area (Heers, 1990). We could find no indication of extramural suburbs c.1300, so we merely used the maximum of all the estimates to reflect any possible spatial growth by this time period.
52. Ghent: The 644 ha area estimates of Russell (1958; 1972) and Chandler & Modelski (1987) refer to the total C14 wall-enclosed area of Ghent (not completed until 1380), which was much larger than the city's settled area and contained expanses of rural land. As such, we use the mean of 80 and 644 as a provisional estimate of the city's actual intramural settled area c.1300 given that about half the land between the inner-city and the full walled zone was occupied (see, e.g., Nicholas, 1987: 67-71; 1992: 130-1, 218; 1997: 185; 1997b: 9, 85-7).

53. Bruges: Russell's (1972) 430 ha estimate and Nicholas' (1997b) estimate both refer to the total C14 wall-enclosed area of Bruges, which was considerably larger than the city's settled area (see, e.g., Nicholas, 1992: 130-1; 1997b: 79). In order to provide a provisional estimate of the intra-mural settled area c.1300, we took the mean of the three estimates because the settled area of Bruges in the early C14 was greater than half of the area between the inner-city and the total wall-enclosed area (see Nicholas, 1997:).
59. Ypres: The estimates from Russell (1958; 1972) correspond to the earlier C12 walled area of Ypres. The detailed map in Jehel & Racinet (1996: 414) indicates that the total walled area of the city was 385 ha, but only about 250 ha of that area was settled.
60. Saint-Omer: Rose (2008: 8) states that Lille and Saint-Omer were the same size (area). Since Lille was roughly 120 ha c.1300 (see below), we have also estimated Lille at 120 ha.
59. Amiens: The map provided by Pounds (2005) shows that the walled area of Amiens measured 100 ha c.1300, suggesting that the 140 ha estimate by Russell (1972) incorporates extramural suburbs.
61. Lille: According to Nicholas (1997b: 72-6; 2003: 70) the extramural suburbs of Lille were abandoned in the 1370, which amounted to one-third of the city's extent. Given that the late C13 wall enclosed some 80 ha, the total settled area c.1300should be about 120 ha. This is probably a conservative figure given the impact of the plague.
62. Liege: Preference given to the measured c.1300 map from Stiennon (1991).
65. Tours: Given that Chandler & Modelski's (1987) estimate specifically excludes two extramural suburbs, we prefer Russell's (1972) larger estimate of 175 ha—which fits nicely with that specification.
71. Albi: Preference given to up-to-date map measurement
72. Perigueux: Preference given to up-to-date map measurement
73. Agen: Preference given to up-to-date map measurement
75. Rodez: Preference given to up-to-date map measurement, which fits with Russell (1958; 1972) specifications
76. Limoges: Preference given to up-to-date map measurement
77. Cahors: Preference given to up-to-date map measurement
78. Tarbes: Preference given to up-to-date map measurement, which fits within Russell's (1972) estimated range
80. Montpellier: Preference given to up-to-date map measurement
81. Narbonne: Preference given to up-to-date map measurement
82. Avignon: Suburban sprawl was a common feature of cities in the French Midi c.1300 (see, e.g, Nicholas, 1997: 184-5; Caille, 1998), so the larger estimate is preferred over the smaller c.1200 estimate.
83. Beziers: Suburban sprawl was a common feature of cities in the French Midi c.1300 (see, e.g, Nicholas, 1997: 184-5; Caille, 1998), so the larger estimate is preferred over the smaller C12 estimate.
85. Arles: Given that Arles was already 40 ha in C12 (Jehel & Racinet, 1996), Russell's (1972) estimate seems too small.
86. Aix-en-Provence: Suburban sprawl was a common feature of cities in the French Midi c.1300 (see, e.g, Nicholas, 1997: 184-5; Caille, 1998), so the larger estimate is preferred over the smaller
90. Carcassone: Suburban sprawl was a common feature of cities in the French Midi c.1300 (see, e.g, Nicholas, 1997: 184-5; Caille, 1998), so the larger estimate is preferred over the smaller
103. Metz: The two estimates are almost identical, so we chose the rounded 160 ha

Northern Italy City Settled Area Estimates (in hectares)

ID#	Name	Nicholas (1997:184-5)	Russell (1972: 44-68)	Russell (1958: 60)	Chandler & Modelski (1987:107-24)	Other Estimates	Final Estimate	Reasoning
104	Bergamo		119				119	Only Estimate
105	Brescia		252				252	Only Estimate
106	Como		96				96	Only Estimate
107	Cremona		165				165	Only Estimate
108	Mantova		215	150 (1242)			215	(see below)
109	Milano		500	314 (C13)	234 (1170)		500	(see below)
110	Monza		56				56	Only Estimate
111	Pavia		158+				158	Only Estimate
112	Padova		350	350 (C14)	76 (1195)	300 (1320) Hyde (1966: 36)	350	(see below)
113	Venezia		324	324 (C14)			324	Only Estimate
114	Verona		150-436			380 (C14) Benevolo (1980:326)	380	(see below)
115	Vicenza		84				84	Only Estimate
116	Genova	150 medieval artisan suburbs walled in C14	293	293 (eC16)			220	Mean
117	Bologna		419	419 (C14)	407 (1206)		419	Mode
118	Faenza		80				80	Only Estimate
119	Ferrara		150		150 (1300)		150	Mode
120	Forli		99				99	Only Estimate
121	Modena		150	150			150	Mode
122	Parma		201	201 (1250)			201	Only Estimate
123	Piacenza	75 (1218-32)	120	345 (C15)		290 (C14) Benevolo (1980:326)	290	(see below)
124	Ravenna		110				110	Only Estimate
125	Reggio Emilia		100	100 (C15)			100	Only Estimate
126	Rimini		48				48	Only Estimate
127	Arezzo		99			107 (C14)	107	(see below)
128	Firenze		630	512 (C14)	512 (1300)	630 (1333) Herlihy (1958: 35)	630	(see below)
129	Pisa	114 (1150), 185 (1300)	185	114 (C13)	114 (1152)	185 (1300) Herlihy (1967: 74)	185	(see below)
130	Pistoia		114	144 (C14)		117 (late C13) Herlihy (1967: 74)	115.5	(see below)

131	Prato		66				66	Only Estimate
132	Siena		90	100 (C14)	50 (1300), 101 (1500s)	180 (C14) Benevolo (1980:326)	180	(see below)
133	Lucca		95	75 (C13)	75 (1200), 95 (1500)	123 (C16) (maps.google.com)	95	(see below)

108. Mantova: Russell's (1972) estimate is specifically for C14, and it makes sense that the settled area will have expanded with population growth between 1242 and c.1300.
109. Milano: Russell's (1972) estimate is specifically for C14, and it makes sense that the settled area will have expanded with population growth between C13 and c.1300.
112. Padova: Considering only the C14 estimates, the map measurement from Hyde (1966: 36) is more recent and authoritative than Russell's *Encyclopedia Italiana*.
114. Verona: Benevolo's (1980) estimate is more recent and authoritative, and fits the range suggested by Russell (1972).
123. Piacenza: Considering only the C14 estimates, the estimate by Benevolo (1980) is more recent and authoritative than the figure suggested by Russell (1972).
127. Arezzo: The estimate by Cherubini (2003: 140) is more recent and authoritative than the figure suggested by Russell (1972).
128. Firenze: Most recent and more authoritative estimates of Herlihy (1958: 35) and Russell (1972) chosen over older and less authoritative sources
129. Pisa: Mode of the estimates ascribed to c.1300 or C14
130. Pistoia: Most recent and more authoritative estimates of Herlihy (1967) corresponds more closely to Russell's (1972) revised C14 estimate
132. Siena: The estimate by Benevolo (1980) is more recent and authoritative than the other figures.
133. Lucca: Lucca's 16th century walls still stand, measuring 123 ha in google maps, casting doubt on Chandler & Modelski's date of 1500. Given the large population of Lucca c.1300, and the addition wall expansions over the course of C13, Russell's (1972) upwards-revised estimate seems the most plausible settled area estimate.

Germany City Settled Area Estimates (in hectares)

ID#	Name	Russell Pre-Plague (1972:80-108)	Chandler & Modelski (1987: 195-209)	Other Sources	Final Estimate	Reasoning
134	Augsburg	178-200			189	Only Estimate
135	Bamberg	70-80			75	Only Estimate
	Bautzen	70			70	Only Estimate
136	Bremen	64+			70	(see below)
137	Dresden	85			85	Only Estimate
138	Erfurt	120	120 (between 1377 and 1400)	>133 walled area (1168) Schlesinger (1967: 261)	133	(see below)
139	Frankfurt am Main	128			128	Only Estimate
140	Goerlitz	72			72	Only Estimate
141	Greifswald	72			72	Only Estimate

142	Halberstadt	77-100			89	Midpoint of Range
143	Hamburg	80-96			88	Midpoint of Range
144	Hannover	54			54	Only Estimate
145	Leipzig	42			42	Only Estimate
146	Luebeck	200			200	Only Estimate
147	Lueneburg	56			56	Only Estimate
148	Mainz	120			120	Only Estimate
149	Noerdlingen	93			93	Only Estimate
150	Nuernberg	138-160			149	Midpoint of Range
151	Paderborn	70			70	Only Estimate
152	Regensburg	95			95	Only Estimate
153	Rostock	98			98	Only Estimate
154	Stralsund	72			72	Only Estimate
155	Stuttgart	50			50	Only Estimate
156	Ulm	66-84			75	Midpoint of Range
157	Wismar	58			58	Only Estimate
158	Worms	170			170	Only Estimate
159	Wuerzburg	72			72	Only Estimate
160	Braunschweig	115			115	Only Estimate
161	Muehlhausen	50			50	Only Estimate
162	Strasbourg	270			270	Only Estimate
163	Basel	49-100			75	Midpoint of Range
164	Konstanz	50			50	Only Estimate
165	Zurich	54-70+			70	(see below)
166	Aachen	175			175	Only Estimate
167	Dortmund	72			72	Only Estimate
168	Koeln	397-401	Rose from 120 to 400(1106-1180)		400	(see below)
169	Muenster	124-156			140	Midpoint of Range
170	Osnabrueck	98			98	Only Estimate
171	Soest	101-120			111	Midpoint of Range
172	Trier	133			133	Only Estimate

136. Bremen: Rounded up to 70 because of the presence of suburbs suggested by Russell (1972)
138. Erfurt: Preference given to Schlesinger (1967) estimate, especially since it suggests greater than 133 ha.
165. Max of Rusell’s (1972) range was chosen given that he suggests the presence of subirbs.
168. Koeln: an even 400 ha was chosen given the general correspondence of the two estimates.

S2.6 Bibliography

Ammann, Hektor. (1969). Wie gross war den mittelalterliche Stadt? In C. Haase (Ed.), *Die Stadt des Mittelalters* (pp. 408-415). Darmstadt: Wissenschaftliche Buchgesellschaft.

Amor, Nicholas. (2011). *Late Medieval Ipswich: Trade and Industry*: Boydell & Brewer Ltd.

Aston, Michael, & Bond, James. (1976). *The landscape of towns*: Dent.

Atkinson, T. D., Hampson, Ethel, Long, E. T. , Meekings, C. A. F., Miller, Edward , Wells, H. B., & Woodgate, G. M. G. (2002). City of Ely: Introduction *A History of the County of Cambridge and the Isle of Ely: Volume 4, City of Ely, Ely, N. and S. Witchford and Wisbech Hundreds*. London.

Bairoch, Paul, Batou, Jean, & Chèvre, Pierre. (1988). *La Population des Villes Européennes: Banque de données et analyse sommaire des réesultats, 800-1850*. Geneva: Librairie Droz.

Baker, N.J., & Slater, T.R. (1992). Morphological Regions in English Medieval Towns. In J. W. R. Whitehand & P. J. Larkham (Eds.), *Urban Landscapes: International Perspectives* London: Routledge.

Balchin, Paul N. (2008). *Urban Development in Renaissance Italy*. Chichester, UK: Wiley.

Barley, M. W. (1976). Town Defences in England and Wales after 1066. In M. W. Barley (Ed.), *The plans and topography of medieval towns in England and Wales* (Vol. 14, pp. 57-70): Council for British Archaeology Research Report.

Barnel, Christine. (1998). Town and Country in Provence: Toulon, Its Notaries, and Their Cities. In K. Reyerson & J. Drendel (Eds.), *Urban and Rural Communities in Medieval France: Provence and Languedoc, 1000-1500* (pp. 239-253). Leiden: Brill.

Barriere, Bernadette. (1984). Limoges. In C. Higounet, J.-B. Marquette & P. Wolff (Eds.), *Atlas historique des villes de France. Par le Centre de recherches sur l'occupation du sol et le peuplement de l'Université de Bordeaux III (E.R.A. 433)*. Paris: Editions du C.N.R.S.

Barron, Caroline. (2000). London 1300-1540. In D. M. Palliser (Ed.), *The Cambridge Urban History of Britain, Volume I: 600-1540* (pp. 395-440). Cambridge: Cambridge University Press.

Benevolo, Leonardo. (1980). *The History of the City*. Cambridge, MA: MIT Press.

Beresford, Maurice Warwick , & St. Joseph, John Kenneth Sinclair (1979). *Medieval England: An Aerial Survey, 2nd Edition*. Cambridge: Cambridge University Press.

Berthe, Maurice, Coquerel, Roland, & Soulet, Jean-Francois. (1982). Tarbes. In C. Higounet, J.-B. Marquette & P. Wolff (Eds.), *Atlas historique des villes de France. Par le Centre de recherches sur l'occupation du sol et le peuplement de l'Université de Bordeaux III (E.R.A. 433)*. Paris: Editions du C.N.R.S.

Biget, Jean Louise. (1983). Albi. In C. Higounet, J.-B. Marquette & P. Wolff (Eds.), *Atlas historique des villes de France. Par le Centre de recherches sur l'occupation du sol et le peuplement de l'Université de Bordeaux III (E.R.A. 433)*. Paris: Editions du C.N.R.S.

Britnell, Richard H. (2000). The Economy of British Towns 1300–1540. In D. M. Palliser (Ed.), *The Cambridge Urban History of Britain, Volume I: 600-1540* (pp. 313-334). Cambridge: Cambridge University Press.

Brodt, Bärbel. (2000). East Anglia. In D. M. Palliser (Ed.), *The Cambridge Urban History of Britain, Volume I: 600-1540* (pp. 639-656). Cambridge: Cambridge University Press.

Buckland, Paul , Magilton, John, & Hayfield, C. (1989). *The Archaeology of Doncaster, 2. The Medieval and Later Town, Part (i)* BAR British Series.

Butler, Lawrence. (1976). The Evolution of Towns: Planned Towns after 1066. In M. W. Barley (Ed.), *The Plans and Topography of Medieval Towns in England and Wales* (pp. 32-47): Council for British Archaeology.

Caille, Jaqueline. (1998). Urban Expansion in Languedoc from the Eleventh to the Fourteenth Century: The Example of Medieval Marseille. In K. Reyerson & J. Drendel (Eds.), *Urban and Rural Communities in Medieval France: Provence and Languedoc, 1000-1500* (pp. 51-73). Leiden: Brill.

Campbell, Bruce MS. (2008). Benchmarking medieval economic development: England, Wales, Scotland, and Ireland, c. 1290. *The Economic History Review, 61*(4), 896-945.

Carpentier, Elisabeth, & Le Mené, Michel. (1996). *La France du XIe au XVe siècle: population, société, économie*: Presses universitaires de France.

Carver, Martin. (1987). *Underneath English Towns: Interpreting Urban Archaeology*. London: B.T. Batsford Ltd.

Cazelles, Raymond. (1972). *Paris de la regne de Philippe Auguste a la mort de Charles V, 1223-1380*. Paris: Hachette.

Chandler, Tertius. (1987). *Four thousand years of urban growth: a historical census*. Lewiston: St. Gavid's.

Cherubini, Giovanni. (2003). *Città communali di Toscana*. Bologna: CLUEB.

Clemens, Jaques. (1985). Agen. In C. Higounet, J.-B. Marquette & P. Wolff (Eds.), *Atlas historique des villes de France. Par le Centre de recherches sur l'occupation du sol et le peuplement de l'Université de Bordeaux III (E.R.A. 433)*. Paris: Editions du C.N.R.S.

De Vries, Jan. (1984). *European Urbanization, 1500-1800*. New York: Routledge.

Dobson, Barrie. (2000). General Survey 1300-1540. In D. M. Palliser (Ed.), *The Cambridge Urban History of Britain, Volume I: 600-1540* (pp. 273-290). Cambridge: Cambridge University Press.

Dury, C. 1986. “” in , 7, p.203. (1986). L’évolution démographique de Tournai au moyen âge *Autor de la ville en Hainaut. Etudes et documents VII* Brussels: Cercle Royal Historique et Archéologiqe d’Ath.

Dyer, Alan. (1995). *Decline and Growth in English Towns 1400-1640* (Vol. 13). Cambridge: Cambridge University Press.

Dyer, Christopher, & Slater, T.R. (2000). The Midlands. In D. M. Palliser (Ed.), *The Cambridge Urban History of Britain, Volume I: 600-1540* (pp. 609-630). Cambridge: Cambridge University Press.

Fenwick, Carolyn C. (1998). *The Poll Taxes of 1377, 1379 and 1381, Part 1: Bedfordshire-Leicestershire*. Oxford: Oxford University Press.

Fray, Jean-Luc. (1997). Nancy. In C. Higounet, J.-B. Marquette & P. Wolff (Eds.), *Atlas historique des villes de France. Par le Centre de recherches sur l'occupation du sol et le peuplement de l'Université de Bordeaux III (E.R.A. 433)*. Paris: Editions du C.N.R.S.

Garrigou-Grandchamp, Pierre, & Mesqui, Jean. (1991). Atlas historique des villes de France. Par le Centre de recherches sur l'occupation du sol et le peuplement de l'Université de Bordeaux III (E.R.A. 433). In C. Higounet, J.-B. Marquette & P. Wolff (Eds.). Paris: Editions du C.N.R.S.

Geremek, Bronislaw. (1987). *The Margins of Society in Late Medieval Paris* (J. Birrell, Trans.). Cambridge: Cambridge University Press.

Goldberg, PJP. (1990). Urban Identity and the Poll Taxes of 1377, 1379, and 1381. *The Economic History Review, 43*(2), 194-216.

Gottfried, R. S. (1982). *Bury St. Edmunds and the urban crisis: 1290-1539*. Princeton: Princeton University Press.

Grantham, G. (2012). France. In H. Kitsikopoulos (Ed.), *Agrarian Change and Crisis in Europe, 1200-1500* New York: Routledge.

Haslam, Jeremy. (2009). Town-plan analysis and the limits of inference: the cases of Bridgnorth and Ludlow, Shropshire. https://jeremyhaslam.files.wordpress.com/2009/12/bridgnorth-and-ludlow-town-plans.pdf

Hatcher, John. (1977). *Plague, population and the English economy, 1348-1530*. London: Macmillan & Co.

Herlihy, D. (1958). *Pisa in the Early Renaissance: A Study of Urban Growth*. New Haven: Yale University Press.

Herlihy, D. (1967). *Medieval and Renaissance Pistoia: The Social History of an Italian Town, 1200–1430*. New Haven: Yale Univerity Press.

Hettinger, Madonna J. (2000). Review of The Poll Taxes of 1377, 1379 and 1381, Part 1: Bedfordshire-Leicestershire by Carolyn C. Fenwick. *Albion, 32*(1), 94-95.

Higounet-Nadal, Arlette. (1984). Perigueux. In C. Higounet, J.-B. Marquette & P. Wolff (Eds.), *Atlas historique des villes de France. Par le Centre de recherches sur l'occupation du sol et le peuplement de l'Université de Bordeaux III (E.R.A. 433)*. Paris: Editions du C.N.R.S.

Hindle, Brian (1990). *Medieval town plans* (Vol. 62): Osprey Publishing.

Hyde, J. K. (1966). *Padua in the Age of Dante*. Manchester University Press.

Jehel, Georges, & Racinet, Philippe. (1996). *La ville médiévale: de l'Occident chrétien à l'Orient musulman, Ve-XVe siècle*. Paris: Armand Colin.

Keene, D. J. (1976). Suburban Growth. In M. W. Barley (Ed.), *The Plans and topography of medieval towns in England and Wales* (pp. 71-82): The Council for British Archaeology.

Keene, Derek, & Rumble, Alexander R. (1985). *Survey of medieval Winchester*. Oxford: Oxford University Press.

Kermode, Jennifer. (1999). Review of The Poll Taxes of 1377, 1379 and 1381: Part 1, Bedfordshire-Leicestershire by Carolyn C. Fenwick. *The Economic History Review, 52*(3), 569-570.

Kermode, Jennifer. (2000). The Greater Towns 1300-1540. In D. M. Palliser (Ed.), *The Cambridge Urban History of Britain, Volume I: 600-1540* (pp. 441-466). Cambridge: Cambridge University Press.

King, David James Cathcart. (1983a). *Castellarium Anglicanum: An Index and Bibliography of the Castles in England, Wales and the Islands, I. Anglesey-Montgomery* (Vol. 1). London: Kraus International Publishers.

King, David James Cathcart. (1983b). *Castellarium Anglicanum: An Index and Bibliography of the Castles in England, Wales and the Islands, II. Norfolk-Yorkshire and the Islands* (Vol. 2). London: Kraus International Publishers.

Kowaleski, Maryanne. (1995). *Local markets and regional trade in medieval Exeter*. Cambridge: Cambridge University Press.

Lartigaut, Jean. (1983). Cahors. In C. Higounet, J.-B. Marquette & P. Wolff (Eds.), *Atlas historique des villes de France. Par le Centre de recherches sur l'occupation du sol et le peuplement de l'Université de Bordeaux III (E.R.A. 433)*. Paris: Editions du C.N.R.S.

Leguay, Jean-Pierre. (2000). Urban Life. In M. Jones (Ed.), *The New Cambridge Medieval History, Volume VI c.1300-1415* (pp. 102-123). Cambridge: Cambridge University Press.

Malanima, P. (2005). Italian Urban Population, 1300-1861.

Miller, John H, & Hatcher, John. (1995). *Medieval England: Towns, Commerce and Crafts, 1083-1348*. London: Routledge.

Nicholas, D. (1987). *The metamorphosis of a medieval city: Ghent in the Age of the Arteveldes, 1302-1390*. Lincoln: University of Nebraska Press.

Nicholas, D. (1992). *Medieval Flanders*. New York: Longman.

Nicholas, D. (1997). *The Later Medieval City, 1300-1500*. London: Longman.

Nicholas, D. (1997). *The Growth of the Medieval City: From Late Antiquity to the Early Fourteenth Century*. London: Longman.

Nicholas, D. (2003). *Urban Europe, 1100-1700*. New York: Palgrave Macmillan.

Nightingale, Pamela. (1996). The Growth of London in the Medieval Economy. In R. H. Britnell & J. Hatcher (Eds.), *Progress and Problems in Medieval England* (pp. 89-106). Cambridge: Cambridge University Press.

Palliser, D.M. (2014). *Medieval York, 600-1540*. Oxford: Oxford University Press.

Palliser, David M. (1992). *The Age of Elizabeth*. London: Longman.

Platt, Colin. (1976). The Evolution of Towns: Natural Growth. In M. W. Barley (Ed.), *The Plans and Topograohy of Medieval Towns in England and Wales* (pp. 48-56): Council for British Archaeology.

Pounds, N. (2005). *The Medieval City*. London: Greenwood Press.

Rawcliffe, Carole. (1999). *Medicine for the Soul: The Life, Death and Resurrection of and English Medieval Hospital. St Giles's, Norwich, c. 1249-1550*: Stroud.

Reyerson, Kathryn. (1998). Urban/Rural Exchange: Reflections on the Economic Relation of Town and Country in the Region of Montpellier before 1350. In K. Reyerson & J. Drendel (Eds.), *Urban and Rural Communities in Medieval France: Provence and Languedoc, 1000-1500* (pp. 253-275). Leiden: Brill.

Rigby, Stephen H. (2010). Urban population in late medieval England: the evidence of the lay subsidies. *The Economic History Review*, *63*(2), 393-417.

Rollo-Koster, Joëlle. (1998). Mercator Florentinensis and Others: Immigration in Papal Avignon. In K. Reyerson & J. Drendel (Eds.), *Urban and Rural Communities in Medieval France: Provence and Languedoc, 1000-1500* (pp. 73-101). Leiden: Brill.

Rose, Susan. (2008). *Calais: An English Town in France, 1347-1558*. Woodbridge: Boydell Press.

Rubin, Miri. (1987). *Charity and Community in Medieval Cambridge*. Cambridge: Cambridge University Press.

Russell, Josiah Cox. (1958). Late ancient and medieval population. *Transactions of the American Philosophical Society, 48*(3), 1-152.

Russell, Josiah Cox. (1972). *Medieval Regions and their Cities*. Bloomington: Indiana University Press.

Rutledge, Elizabeth. (1988). Immigration and population growth in early fourteenth-century Norwich: evidence from the Tithing Roll. *Urban History, 15*, 15-39.

Rutledge, Elizabeth. (1995). Landlord and tenants: housing and the rented property market in early fourteenth-century Norwich. *Urban History, 22*(1), 7-24.

Rutledge, Elizabeth. (2004). Norwich before the Black Death/Economic life. In C. Rawcliffe & R. Wilson (Eds.), *Medieval Norwich* (pp. 157-188). London: Bloomsbury.

Schofield, J., & Vince, A. G. (2003). *Medieval towns: the archaeology of British towns in their European setting*. London: A&C Black.

Stabel, Peter. (1997). *Dwarfs among giants : the Flemish urban network in the late Middle Ages*. Leuven Garant.

Steane, John. (2014). *The archaeology of medieval England and Wales*. London: Routledge.

Stephenson, Carl. (1937). *Borough and town: a study of urban origins in England*. Cambridge, Mass.: The Mediaeval Academy of America.

Stiennon, Jacques. (1991). *Histoire de Liège*: Privat.

Suau, Bernadette. (1983). Rodez. In C. Higounet, J.-B. Marquette & P. Wolff (Eds.), *Atlas historique des villes de France. Par le Centre de recherches sur l'occupation du sol et le peuplement de l'Université de Bordeaux III (E.R.A. 433)*. Paris: Editions du C.N.R.S.

van Bavel, B. (2010). *Manors and Markets: Economy and Society in the Low Countries, 500-1600*. Oxford: Oxford University Press.

Wickham, Chris. (2015). *Medieval Rome. Stability and Crisis of a City, 900-1150*. Oxford: Oxford University Press.
